# Coordination mechanisms for COVID-19 in the WHO Regional office for Africa

**DOI:** 10.1186/s12913-022-08035-w

**Published:** 2022-05-28

**Authors:** Nsenga Ngoy, Boniface Oyugi, Paul O. Ouma, Ishata Nannie Conteh, Solomon Fisseha Woldetsadik, Miriam Nanyunja, Joseph Chukwudi Okeibunor, Zabulon Yoti, Abdou Salam Gueye

**Affiliations:** 1 World Health Organisation, Regional Office for Africa, Emergency Preparedness and Response Programme, Brazzaville, Congo; 2 World Health Organisation, Regional Office for Africa, Emergency Preparedness and Response Programme, Nairobi, Hub Kenya

**Keywords:** Coordination Mechanism, COVID-19, Health Emergencies, Pandemic Management, WHO Regional Office for Africa

## Abstract

**Aim:**

This study describes the coordination mechanisms that have been used for management of the COVID 19 pandemic in the WHO AFRO region; relate the patterns of the disease (length of time between onset of coordination and first case; length of the wave of the disease and peak attack rate) to coordination mechanisms established at the national level, and document best practices and lessons learned.

**Method:**

We did a retrospective policy tracing of the COVID-19 coordination mechanisms from March 2020 (when first cases of COVID-19 in the AFRO region were reported) to the end of the third wave in September 2021. Data sources were from document and Literature review of COVID-19 response strategies, plans, regulations, press releases, government websites, grey and peer-reviewed literature. The data was extracted to Excel file database and coded then analysed using Stata (version 15). Analysis was done through descriptive statistical analysis (using measures of central tendencies (mean, SD, and median) and measures of central dispersion (range)), multiple linear regression, and thematic analysis of qualitative data.

**Results:**

There are three distinct layered coordination mechanisms (strategic, operational, and tactical) that were either implemented singularly or in tandem with another coordination mechanism. 87.23% (*n* = 41) of the countries initiated strategic coordination, and 59.57% (*n* = 28) initiated some form of operational coordination. Some of countries (*n* = 26,55.32%) provided operational coordination using functional Public Health Emergency Operation Centres (PHEOCs) which were activated for the response. 31.91% (*n* = 15) of the countries initiated some form of tactical coordination which involved the decentralisation of the operations at the local/grassroot level/district/ county levels. Decentralisation strategies played a key role in coordination, as was the innovative strategies by the countries; some coordination mechanisms built on already existing coordination systems and the heads of states were effective in the success of the coordination process. Financing posed challenge to majority of the countries in initiating coordination.

**Conclusion:**

Coordinating an emergency is a multidimensional process that includes having decision-makers and institutional agents define and prioritise policies and norms that contain the spread of the disease, regulate activities and behaviour and citizens, and respond to personnel who coordinate prevention.

**Supplementary Information:**

The online version contains supplementary material available at 10.1186/s12913-022-08035-w.

## Introduction

Coronavirus Disease 2019 (COVID-19) has now spread to all countries in the African continent, the caseload reports from the member states indicate that the pandemic has spread at a much slower rate on the continent than in the rest of the world, contrary to previous predictions [1]. The African continent is characterised by heterogeneity in culture, demography, disease patterns, economy, geography, language, politics, and social equity [1]. Several reasons have been postulated for the slow rate, including the role of aridity and temperature in transmission, demographic characteristics (distribution of age), and the difference in identification of cases, and death detection capacity [2–7], and the possible contribution of pre-existing immunity from other viral infections [8]. Others have indicated that the numbers are due to the underestimation of the true magnitude of the pandemic resulting from weak surveillance systems [9, 10].

Predictions about the African continent in the form of initial modelling the COVID-19 projections and the pandemic evolutions came from more so entities outside of the continent who were trying to fit their other global experiences to Africa, without fully appreciating the heterogeneity diversity across the continent [3, 11–17]. These differences exist at the country national and subnational levels and extend into areas of attainment of International Health Regulation capacity, health systems, and social services, and they affect each country’s response to the COVID-19 pandemic [18, 19]. Although all the above-mentioned factors may have contributed to the lower pace of transmission, the rapid and relatively early and timely adequate response has likely contributed. One of the key determinants of the emergency response is the coordination mechanisms. This paper describes the coordination mechanisms for COVID response in countries of the African region (AFRO) of the World Health Organization (WHO).

Coordination is defined as the management process to ensure integration (unity) of effort. It relates ‘primarily to resources and operates vertically (within an organization) as a function of the authority to command and horizontally (across organizations) as a function of the authority to control how different organizations (public or private) or parts of the same organization work or act together to achieve a common objective ’[20] (pg. 12). The WHO defines multisectoral coordination as a ‘deliberate collaboration between stakeholders from multiple and diverse sectors and disciplines towards the shared goal and enhanced health emergency preparedness and response’ and whose effectiveness depends on political, economic, and social factors [21] (pg.2). Coordination mechanism in emergency response is meant to maintain and establish a smooth information and decision-making flow as well as an effective working relation between various entities involved in the emergency response [22]. The coordination of response is demanding as it involves the interactions factors that characterise emergency such as sudden and unexpected events; great uncertainty; severe resource shortages; high amounts of time pressure and urgency; large-scale impact and damage; the risk of possible mass casualty; and the disruption of infrastructure support necessary for coordination. Further, this is complicated by factors such as multi-authority and massive personal involvement, infrastructure interdependencies, conflict of interest cases, and the high demand for timely information [23]. Because of the complexity of number of entities involved both strategically, operationally, administratively, and geographically, and because of the often-changing dynamic of the emergency which is often time-sensitive, Shan and Yan [22] recognized that coordination is one of the most challenging aspects of the emergency response.

Previous research work particularly in response to Haemorrhagic fevers in Africa has shown that the needed swift public health response coordinated by ‘international agencies, funding organisations, and most importantly, the national health institutions at the district, local government, state, and regional levels to curb the recent outbreaks’ is an essential component of reducing the response which has been lacking in the previous management of Haemorrhagic fevers in Africa [24] (pg. e496). For instance, coordination has been noted as an essential component of the quality of emergency response management particularly for previous responses of Ebola and Marburg [25]. The coordination of the 2014-2016 Ebola outbreak in West Africa was characterised by the unpreparedness and poor coordination of the response at the national and regional levels, leading to its fast spread [26]. Learning from the past lessons of response, the subsequent coordination efforts in the 2018–2020 Ebola outbreak in the Democratic Republic of the Congo (which faced additional challenges due to armed rebel groups being at the epicentres of the epidemic) [27] and in Guinea in 202 1[28] resulted in strengthened inter-state coordination. The coordination mechanism involved the WHO regional office for Africa and Africa Union (AU) member states through the Africa Centres for Disease Control and Prevention (Africa CDC). The WHO’s prompt declaration of a Public Health Emergency of International Concern (PHEIC) shepherded an effective coordinated response to contain the epidemic. It enhanced the development and implementation of a preparedness and readiness plan focused on building and sustaining resilient capacities to prevent, detect, and respond to future outbreaks following the International Health Regulations (IHR) [27].

Emerging lessons from the different countries’ coordination of the COVID-19 response has shown the implementation of several strategies to mitigate the impact of the response. For example, the creation of a social media platform to create an information-sharing network accelerated some government response and recovery efforts [29]. Else ways, the tailoring of the response based on institutional and political contexts such as either such as either centralisation or the use of local initiative in responses influenced the response outcomes [30]. However, challenges of insufficient specifications on coordinating emergency responses, especially those involving cross federal, state, and local governments has been shown to lead to challenges in establishing context-tailored and effective coordination mechanism resulting in poor coordination, blurred lines of authority, and communication breakdowns [31]. Both organisations and individuals make decisions to achieve the uninterrupted operation of sequential tasks during emergency response, and the success of the response is largely affected by effective coordination and collaboration. Response to public health emergencies (PHE) requires a collective endeavour through inter-organisational networks [32], but PHEs are primarily addressed by country’s public health system whose structure significantly varies from one country to another within the AFRO region [33, 34]. National PHEs require considerable effort to collect, assemble, analyse, and make health information available to communities through coordination and collaboration at different levels of government [31]. In overall, decisive leadership has been shown as an essential ingredient in the COVID-19 response [35].

The main responsibility of the coordination mechanisms is for directing public health response in the jurisdiction that is affected and coordinating the efforts of all health stakeholders. More importantly, early actions and enhanced coordination mechanisms both at country and regional levels are critical to lowering or at least slowing down the spread of a pandemic. However, these coordination mechanisms have not been fully evaluated in a region-wide pandemic in Africa, hence, this paper a) describes the coordination mechanisms that have been used for management of the COVID-19 pandemic in the WHO AFRO region; b) relates the patterns of the disease to coordination mechanisms established at the national level, and c) documents emerging themes on coordination through a retrospective description of the emerging themes and the commonalities between coordination strategies.

## Methodology

### Design

The study employed mixed methods approach that utilised both quantitative and qualitative data gathered from a review of WHO AFRO countries’ coordination mechanisms for COVID-19 response through a retrospective policy tracing [36]. The analysed COVID-19 coordination mechanisms covered the period from March 2020 (when first cases of COVID-19 in the AFRO region were reported) to the end of second wave in the different countries (the end dates of the first and second waves in the countries varied). The combination of the analysis of quantitative and qualitative methods complemented each other and allowed for the exploration not only of ‘what’ questions but of ‘how’ and ‘why’ questions [37, 38]. The results of qualitative and quantitative data (as described below) were analysed separately, compared or combined where applicable to get a complete understanding of the COVID-19 coordination mechanisms.

### Analytical framework

**Fig. 1:**
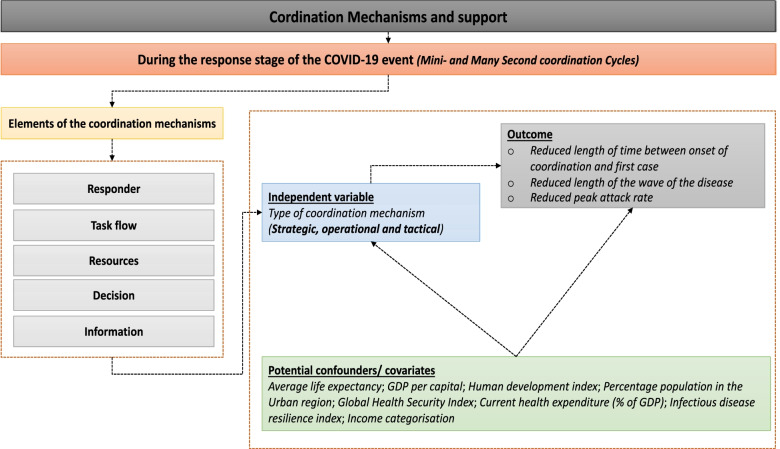
A priori conceptual framework (source Chen et al. [23] and document reviews)

According to Chen et al. [23], the coordination of emergency management has three main stages: the pre, during and post-incident coordination each comprising of five elements namely: task flow, resources, decisions, responder, and information. The *task flow* focuses on the tasks and interdependent relationships; *resources* focus on resource utilisation management and dependencies; *information* focuses on task-critical information collection, analysis, and distribution; *decision* focuses on the decision roles, rules and structure; while *responder* focuses on the relationships, team-think, group dynamics, and organisational dynamics*.* In this review we analysed the countries’ coordination mechanisms during the response stage of the COVID-19 event focusing on the five elements. The analytical framework follows the conceptual framework outlined (Fig. 1).

Based on the synthesis of the elements of coordination, we defined coordination mechanisms based on three levels – strategic, operational, and tactical, which depending on the country, can either be implemented singularly or in tandem with another coordination mechanism. First, is the strategic coordination having two levels: level 1 coordination (led by the President, Prime ministers, or Vice presidents of different countries) or level 2 coordination (led by different ministerial task forces or cabinet officials appointed by the presidents). Second is the operational coordination using functional PHEOCs activated for the response. The activation of the national PHEOC is followed by the COVID-19 readiness and response Incident Management System (IMS) which oversees the preparedness and response mechanisms for any public health emergency. This level also consists of technical expertise covering different areas such as case management, logistics, partnerships, laboratory, surveillance, and information management. PHEOCs maintain situational awareness across the system at the operational level, and some mobilised and deployed local health care resources. Third is the tactical coordination which involves the decentralisation of the operations to the at the local/grassroot level/district/ county levels. According to the PHEOC handbook [39] an effective coordination mechanism requires all three levels working in tandem and a lack of any of the levels more so in a pandemic may lead to an inadequate response.

Coordination mechanisms exist to employ technical expertise to limit the introduction of the infection in the community. Additionally, it limits the spread and infectiousness of the disease in the event of local transmission. This is done through the use of evidence and multisectoral mechanisms. We, therefore, assumed that an optimally functioning coordination mechanism reduces the length of time between the onset of coordination and the first case, the length of the wave of the disease, and peak attack rate as proxies.

The existence of coordination mechanisms is however also driven by other factors such as income status of a country as this affects ability to allocate resources and prioritise interventions, infectious disease resilience index which shows how a country deals with historical infectious diseases and may affect quality of coordination mechanisms. Additionally, Global Health Security Index (GHSI) seeks to illuminate preparedness and capacity gaps to increase political will and financing to fill them at the national and international levels, which would determine the coordination mechanisms [40]. However, other factors such as testing capacity, population density, and institution of social measures were not included in the analysis as they likely exist in the causal pathway between coordination and epidemiology of the disease. Essentially, decisions on what to do including public health and social measures and how testing is conducted are informed by the coordination teams. We additionally excluded population density but instead used proportion of total population in urban areas which is a better indicator of transmission.

### Data sources

Data utilised in this review was extracted in three steps using complementary methods. The first steps involved data collection through retrospective policy tracing using a comprehensive media and government policy reports review and document review in the first phase. A chronological examination of the published media the 47 countries COVID-19 response strategies, plans, regulations, press releases, government websites, grey and peer-reviewed literature for information regarding the coordination structures was conducted (Table 1). This step was to identify the elements of the coordination mechanism (Fig. 1). The document review brings evidence on policy formulation or implementation and highlights the policy window through which issues gets/ got to the fore of the policymakers’ agenda [36]. The review of the grey and peer-reviewed literature, strategies, plans, regulations, press releases, official government letters, decrees, and communications from the government websites and media reports included information published in English, French, Spanish and Portuguese (official languages in the member states of the WHO African regions). Out of 456 articles reporting COVID-19 coordination mechanisms in all the 47 countries, 103 were extracted and utilised as they showed how the mechanisms worked (Additional file 1). Each document was then reviewed and summarised in a word document (per country) that captured information on coordination at the national level and the county/ district/ local regions (governance, roles, and information flow); when coordination started; and the changes/ gaps and lessons learnt (see extraction sheet in Additional file 1). The review of documents was conducted independently by two different reviewers from the research team to achieve consensus.

**Table 1:** Document review and sources of data

Component extracted	Source
Coordination mechanisms	COVID-19 response strategies, plans, regulations, press releases, government websites, other websites, grey and peer-reviewed literature
Average life expectancy	United Nations Development Programme (UNDP) (http://hdr.undp.org/en/countries) [41]
GDP per capita	The World Bank Database (https://data.worldbank.org/) [42]
Income categorisation	The World Bank Database (https://data.worldbank.org/) [42]
Human development index	United Nations Development Programme (UNDP) (http://hdr.undp.org/en/countries) [41]
Percentage population in the Urban region	United Nations Development Programme (UNDP) (http://hdr.undp.org/en/countries) [41]
Global Health Security Index	Global Health Security Index (GHSI) (https://www.ghsindex.org/) [40]
Current health expenditure (% of GDP)	Zhang et al. [43]
Infectious disease resilience index	Zhang et al. [43]
Attack rate	WHO COVID-19 dashboard [44]
Length of the wave	WHO COVID-19 dashboard [44]

The second step involved a confirmation of any unofficial sources and focussed on filling the gaps, especially for countries where little or no information had been extracted. Any unofficial data sources were verified by direct communication with the Member States through the WHO focal points or other official sources before inclusion in the extraction datasheet.

The third step involved the transformation of the information gathered from the above two steps into independent variable (Fig. 1); and gathering and extracting additional information per country (on outcomes and potential confounders/ covariates) using sources of the data as shown in Table 1 into an Excel file. The summary of the characteristics of the countries in the AFRO region are shown in Additional file 2*.*

### Data management and analysis

In overall all the data management and analysis in this review were analysed and fitted through thematic framework approach guided by the elements of coordination mechanisms (Fig. 1). In step one of the analyses, the qualitative information extracted from phase one and two of the data sources (described above) were analysed thematically. The framework guided the analysis processes and patterns of convergence were assessed by drawing on techniques of the constant comparison method.

In step two, all information for each country that extracted into an Excel file database were coded as shown in Table 2. Data was cleaned and sorted using the excel filter function. The data was then analysed using Stata (version 15). Descriptive statistical analysis was carried to describe the demographic characteristics of the countries and the coordination mechanisms and was presented in frequency, percentages, and measures of central tendencies (mean, SD, and median) and measures of central dispersion (range).

**Table 2:** Definition and measurement of variables used in multilinear regression model

Variable definition	Measurements
***Outcome variables***	
Length of time between onset of coordination and first case	Continuous
Length of the wave of the disease	Continuous
Peak attack rate	Continuous
***Independent variables***	
Layered coordination mechanism	0 – Strategic and tactical
	1 – Strategic, operational, and tactical
	2 – Strategic and operational
	3 – Operational or tactical
Income categorisation	1 – High income
	2 – Upper middle income
	3 – Lower middle income
	4 – Low income
Average life expectancy	Continuous
GDP per capita	Continuous
Human development index	Continuous
Percentage population in the Urban region	Continuous
Global Health Security Index	Continuous
Current health expenditure (% of GDP)	Continuous
Infectious disease resilience index	Continuous

We hypothesised that the type of coordination mechanism plays a role in reducing the length of time between onset of the disease and the institution of coordination mechanism; it reduces the length of the wave of the disease and reduces the attack rate at the peak of the wave. These assumptions were underpinned by literature on the different characteristics that elucidate the impact of coordination mechanism and for which we curated a conceptual model (Fig. 1).

To test the relationship, we used multiple linear regression approach to estimate the relationship between the type of coordination mechanism and the length of time between onset of the disease and the institution of coordination mechanism, the length of the wave of the disease and the attack rate at the peak of the wave. The starting basic OLS regression model is denoted as:

1 $${y}_{it}={\beta}_0+{\beta}_1{Cordination}_{it}+{X}_{it}{\beta}_2+{\varepsilon}_{it}$$

Where, *y*_*it*_ is the outcome (length of time between onset of coordination and first case; length of the wave of the disease; and peak attack rates) which are continuous variables for each of the country *i,* at time *t; β*_0_ is the intercept; *β*_1_ is the slope associated with the independent variable; *Cordination*_*it*_ is the variable type of coordination mechanism (in categorical form); which was formulated to estimate the effects of coordination mechanisms in outcome variables with OLS in time approach using robust standard errors; *X*_*it*_ is the vector of the included characteristics (Average life expectancy, GDP per capita, Human development index, Percentage population in the Urban region, Global Health Security Index, Current health expenditure (% of GDP), Infectious disease resilience index, and Income categorisation), and *ε*_*it*_ is the intercept. The definitions of the variables are shown in Table 2.

Before fitting the regression model, we conducted Pierson’s correlation coefficient test amongst the variables (Additional file 3) to test for collinearity and remove confounding variables*.* We removed the income categories variables of the countries because it was highly correlated with GDP per capita (− 0.8794), and human development index (− 0.8684). The full iteration is shown in the SI.

This study did not require ethical approval for the study protocol and data collection as all the data utilised were publicly available.

## Results

### Responder

#### Characteristics of the countries analysed

A summary of the countries characteristics is shown in Table 3. The average life expectancy in the 47 AFRO countries is 63.29 years with Central African Republic having the lowest 53.3 while Algeria has the highest at 76.9 years; the average GDP per capita is 5537.38; human development index 0.55; Global Health Security Index 31.26 and Infectious disease resilience index of 0.30. The country with the highest percentage population in the urban region is Gabon at 89.7 while the lowest is Burundi at 13.4% and the country with the highest current health expenditure (% of GDP) is Sierra Leone at 16.06% and the lowest is Congo at 2.14%, while the average stands at 5.58%. In overall, the majority of the countries (53.19%) are categorised as low-income countries.

**Table 3:** Summary Country characteristics and coordination methods

**County characteristic variables (continuous)**	***n*** **= 47**				
	**Mean**	**SD**	**Median**	**Min**	**Max**
Average life expectancy	63.29	5.37	63.4	53.3	76.9
GDP per capita	5537.38	6248.96	3081	752	29,056
Human development index	0.55	0.54	0.10	0.39	0.80
Percentage population in the Urban region	44.42	18.25	43.1	13.4	89.7
Global Health Security Index	31.26	7.58	31.1	16.2	54.8
Current health expenditure (% of GDP)	5.58	2.56	5.11	2.14	16.06
Infectious disease resilience index	0.30	0.14	0.270681	0.00006	0.69729
**Coordination variables (categorical)**	***n*** **= 47**				
	**Categories**		**Frequency (n)**	**Percentage (%)**	
Income categorisation	High income		1	2.13	
	Upper middle income		7	14.89	
	Lower middle income		14	29.79	
	Low income		25	53.19	
Initiated strategic coordination	Yes		41	87.23	
	No		6	12.77	
Initiated operational coordination	Yes		28	59.57	
	No		19	40.43	
Initiated tactical coordination	Yes		15	31.91	
	No		32	68.09	
Implemented PHEOC before COVID-19	Yes		38	80.85	
	No		9	19.15	
Functional PHEOC before COVID-19	Yes		26	55.32	
	No		21	44.68	
Layered coordination mechanism (Fig. 2)	Both strategic and tactical		10	23.26	
	Strategic, operational, and tactical		10	23.26	
	Both strategic and operational		16	37.21	
	Only operational or tactical		7	16.28	

#### Coordination mechanisms that have been used for management of COVID-19 pandemic in WHO AFRO region

**Fig. 2:**
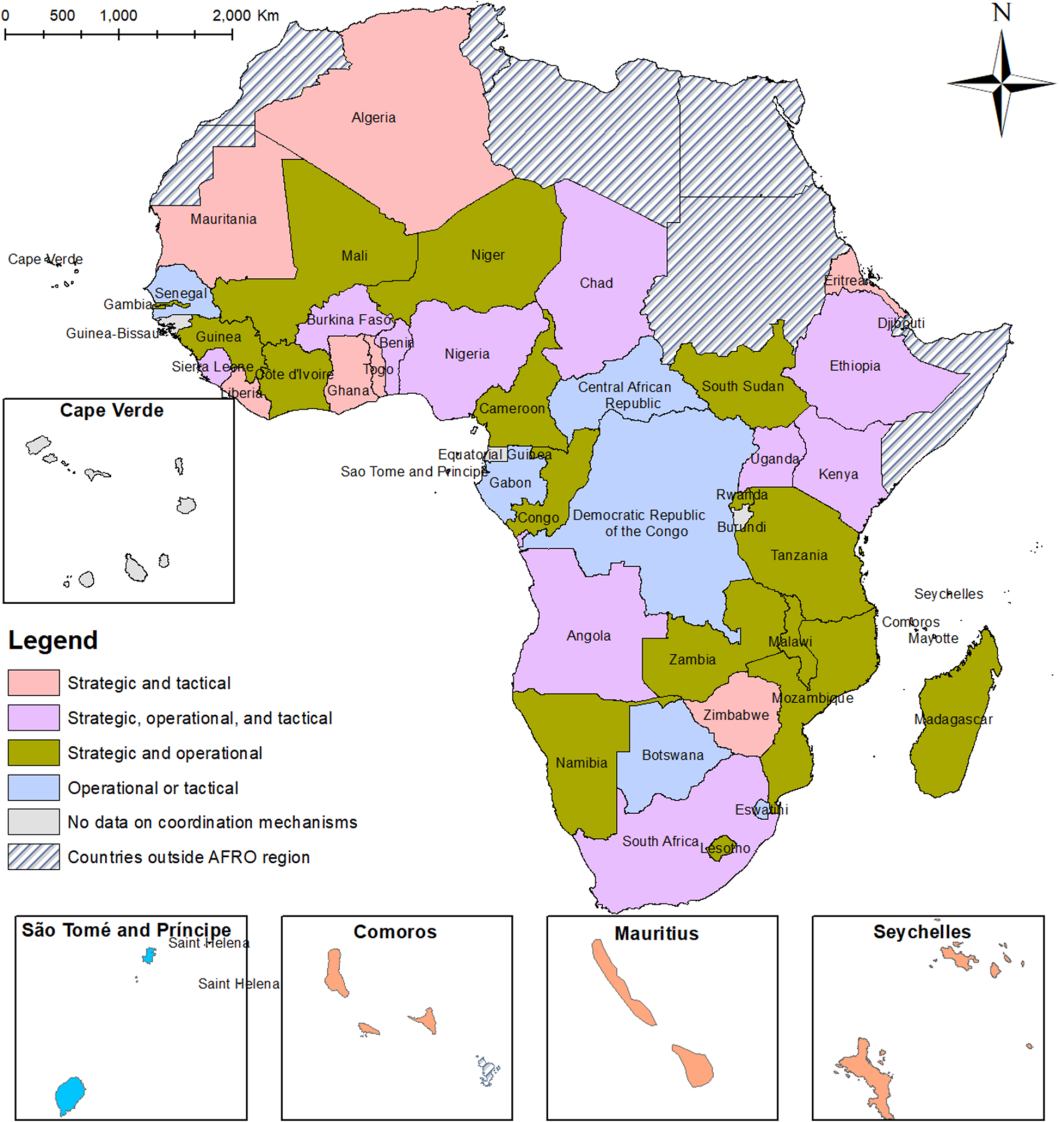
The layered coordination mechanisms in Africa continent

Our finding shows three distinct layered coordination mechanisms (strategic, operational, and tactical) (Fig. 2) were either implemented singularly or in tandem with another coordination mechanism. Most of the countries (*n* = 41, 87.23%) initiated strategic coordination. For example, in Côte d’Ivoire, the National Steering Committee provided both strategic and policy guidance (chaired by the Prime Minister) and the Steering Committee/ secretariat of the Steering Committee led by the Ministry of Sanitation, and Public Health provided both strategic and policy guidance, and oversight for the Governments’ emergency response with a crisis committee instituted on need [45]. Tanzania had three committees (in different coordination levels) that were tasked with leading the fight against the COVID-19 pandemic though late in the pandemic [46]. In Mauritania, both strategic and operational work was committee led [47].

59.57% of the countries initiated some form of operational coordination. Some of countries (55.32%) provided operational coordination using functional PHEOCs which were activated for the response. 31.91% of the countries initiated some form of tactical coordination which involved the decentralisation of the operations to the at the local/grassroot level/district/ county levels *(*Table 3*).*

#### Relationship between coordination mechanisms and epidemiological outcomes

The relationship findings are presented in Tables 4, 5 and 6. Our findings show that there is no statistically significant difference in the relationship between the type of coordination mechanism applied in the country with the length of time between onset of coordination and first case (in days); the length of wave 1; attack rate at wave 1 Peak; and attack rate at wave 2 Peak. However, there is a statistically significant difference in the coordination mechanism applied in the country with the length of wave 2. For instance, there are 69.73 days more on average in the length of waves 2 among countries that use coordination mechanism 1 (strategic, operational, and tactical) as compared to coordination mechanism 0 (only Strategic and tactical); and there are 66.26 days less on average in the length of waves 2 among countries that use coordination mechanism 3 (only strategic and operational) as compared to coordination mechanism 0 (only Strategic and tactical) (Table 6).

**Table 4:** Relationship between length of time between onset of coordination and first case (in days) and coordination mechanism

Length of time between onset of coordination and first case (in days)	Coef.	***p*** value	[95% Conf. Interval]	
			Lower bound	Upper bound
**Coordination mechanism (Ref: 0)**				
1	− 78.68	0.331	− 256.85	99.49
2	−22.88	0.649	− 136.74	90.98
3	− 143.94	0.211	− 391.41	103.53
**Average life expectancy**	6.92	0.325	−8.55	22.40
**GDP per capita**	0.02	0.137	−0.01	0.06
**Human development index**	− 1084.56	0.280	− 3277.21	1108.08
**Percentage population in the Urban region**	0.78	0.757	−4.94	6.50
**Global Health Security Index**	1.81	0.757	−11.45	15.06
**Current health expenditure (% of GDP)**	4.47	0.739	−26.08	35.02
**Infectious disease resilience index**	− 255.05	0.557	− 1232.03	721.93
**_cons**	71.20	0.876	− 968.57	1110.97

**Table 5:** Relationship between Length of wave 1 and Attack Rate at wave 1 Peak with coordination mechanism

	Length of wave 1				Attack Rate at wave 1 Peak			
	Coef.	***p*** value	[95% Conf. Interval]		Coef.	***p*** value	[95% Conf. Interval]	
			Lower bound	Upper bound			Lower bound	Upper bound
**Coordination mechanism (Ref: 0)**								
1	96.97	0.180	−48.11	242.05	828.22	0.235	− 572.91	2229.35
2	72.32	0.272	− 60.87	205.50	173.64	0.743	− 905.69	1252.96
3	91.01	0.152	−36.12	218.14	531.77	0.46	− 928.77	1992.31
**Average life expectancy**	2.76	0.438	−4.49	10.01	−2.11	0.967	− 107.25	103.02
**GDP per capita**	0.00	0.645	−0.01	0.01	0.00	0.963	−0.16	0.16
**Human development index**	142.00	0.707	− 631.08	915.08	− 640.44	0.915	−12,815.66	11,534.78
**Percentage population in the Urban region**	0.43	0.458	−0.76	1.63	38.28	0.159	−16.06	92.61
**Global Health Security Index**	−0.52	0.748	−3.86	2.81	139.02	0.105	−31.05	309.09
**Current health expenditure (% of GDP)**	1.43	0.758	−8.10	10.96	86.89	0.568	− 222.06	395.83
**Infectious disease resilience index**	− 149.19	0.432	− 535.75	237.38	3715.71	0.115	− 965.05	8396.48
**_cons**	− 208.81	0.506	− 848.76	431.15	− 7097.79	0.171	−17,456.30	3260.73

**Table 6:** Relationship between Length of wave 2 and Attack Rate at wave 2 Peak with coordination mechanism

	Length of wave 2				Attack Rate at wave 2 Peak			
	Coef.	***p*** value	[95% Conf. Interval]		Coef.	***p*** value	[95% Conf. Interval]	
			Lower bound	Upper bound			Lower bound	Upper bound
**Coordination mechanism (Ref: 0)**								
**1**	**69.73**	0.043*	**2.39**	**137.07**	1522.64	0.189	− 800.70	3845.97
2	0.08	0.999	−93.72	93.88	912.62	0.325	− 962.40	2787.65
3	−66.26	0.248	− 182.52	50.01	1077.56	0.367	− 1339.75	3494.86
**Average life expectancy**	−4.79	0.278	−13.76	4.18	−21.93	0.764	−171.04	127.17
**GDP per capita**	0.00	0.336	−0.01	0.00	0.00	0.974	−0.24	0.24
**Human development index**	751.17	0.189	− 401.49	1903.83	491.93	0.956	−17,921.64	18,905.51
**Percentage population in the Urban region**	−0.57	0.468	−2.18	1.04	61.48	0.179	−30.25	153.22
**Global Health Security Index**	−5.93	0.060	−12.13	0.28	220.41	0.107	−51.52	492.35
**Current health expenditure (% of GDP)**	1.99	0.740	−10.36	14.34	148.86	0.548	− 354.81	652.52
**Infectious disease resilience index**	− 122.31	0.590	− 589.26	344.63	5873.88	0.112	− 1472.15	13,219.92
**_cons**	194.62	0.384	− 262.26	651.50	−11,254.50	0.181	−28,111.75	5602.75

#### Role of different stakeholders

Overall, this study’s findings show that different stakeholders played various roles right from technical, financing, implementation, and even advisory. In all the countries, the government officials or government-led taskforces coordinated the preparedness and response on logistics, funds raising and management, healthcare data collection and analysis. Additionally, they played the main function of official communications and provided regular updates on the COVID-19 situation. Besides, the government entities helped guide the health system’s response, inform broader political decisions and directed the health system’s response to the pandemic. In Madagascar, for example, a government military platform was created to support the digitalization of cases and hospital bed attribution [16].

Other players were well-renowned experts in different fields who advised governments on the direction of the response. For instance, in DRC, Professor Jean-Jacques Muyembe, the eminent Congolese virologist who discovered the Ebola virus in 1976, led the national response to Ebola and COVID-19 (which occurred concurrently) [48].

Furthermore, the Development Partners (DPs) and United Nations (UN) agencies provided financial and technical support for testing capacity training programs, enhancing contact tracing, and assistance to vulnerable populations. UN/WHO provided extensive technical assistance in coordination, risk communication and community engagement, surveillance and Rapid Response Teams (RRTs), and Points of Entry (POE). Others such as UNICEF, UNESCO and UNDP were the main lead agencies for social protection interventions.

Multilateral governments and private partner companies (such as ExxonMobil, Chevron and Jack Ma Foundation) were also essential in coordinating the response, for instance, by donating personal protective equipment funding training programs, among others [49]. Cuba, for instance, doctors and medical supplies to Angola and Qatar and Portugal provided personal protective gear.

### Task flow

#### Decentralisation strategies played a key role

The findings revealed that the decentralised strategies – involving coordination structures to the subnational levels – played a significant role in the countries, but they were uniquely applied by each country. For instance, in Cameroon, in consultation with civil society actors, parliamentarians and development partners assisting in the pandemic response, the government actioned a series of measures to decentralise the pandemic management to the regional/local levels, such as the establishment of treatment centres for COVID-19 patients in all the 10 regional capitals [50]. The strategy was like that applied in Kenya, where the Council of Governors (COG) established the cross-sector COVID − 19 Secretariat to coordinate counties’ response and recovery strategy [51]. The aim was to enhance the operations of pandemic management, leaving the central government to focus on strategy development and resource mobilisation.

In some countries, such as Burkina Faso, the decentralisation strategy sought to enhance the motivation and morale of HCWs to manage COVID-19 cases. However, the motivation payments were only allowable for HCWs involved in COVID-19 treatment leaving out critical players such as community health workers who played a significant role but are not integrated into COVID-19 purchasing arrangements hence a gap in motivation and satisfaction [52]. However, to enhance the coordination of the resources of commodities and Personal Proctective Equipment (PPE) to the regional level; and treatment of COVID-19 to all health facilities, there was well-decentralised strategies such as COVID-19 testing (rapid diagnostic tests) to district hospitals (CMA) [52].

In two countries, Ethiopia and South Africa, the decentralisation of response to the Sub-national (regional) level took a synergistic approach. For instance, in South Africa, the decentralisation of COVID response to provinces, districts, and sub-districts included creating provincial incident management teams (IMTs) akin to those at the national level, which comprised a variety of workstreams that worked in synergy [53]. Similarly, in Ethiopia, the humanitarian actions were coordinated by the established EOC, and national and regional task forces were established in all regions [54].

For other countries, the strategy involved working with already established community health strategies and structures. For example, with the support of the WHO, the Ministry of Health (MOH) Angola deployed public health experts from Luanda to other provinces [49], while in Benin, there was a multidisciplinary community brigade that was operationalised in the 77 communes of Benin to identify problem targets and raise their awareness [55]. Similarly, in Botswana, the community-based surveillance strategy was introduced to work with local organisations and local community members who knew best what the communities need [56]. In Eswatini, the regional committees were chaired by Regional Administrators and various Sector Committees who coordinated the response and implementation plan in the regions [57]. Nigeria specifically used the hot-spot strategy for decentralisation that was mainly actioned for hot spot areas [58].

The decentralisation of case management activities in hospital and community centres relied on the already existing structure used during the Ebola outbreak in Sierra Leone [59]. The implementors reactivated the community sensitisation structures where the MOH and Sanitation officials visited and informed several community leaders nationwide about COVID-19.

#### Use of innovative projects in coordination

As part of the multifaceted and multisectoral approach to combatting and containing the COVID-19 pandemic in the region, our findings show that countries applied innovative governance and operational strategies. Notable strategies are targeted at ensuring that there is continuity of essential services, cross-border movement strategies, coordinating the aspects of Risk Communication and Community Engagement (RCCE), and monitoring the progress and relaxation of Public health and social measures (PHSMs).

Specifically, countries such as Rwanda [60] and Ghana [61] have disseminated public information through drones and used robots for screening and inpatient care. Others have conducted official communications through social media platforms to combat misinformation and mobilise a cohesive response from the population. Examples include mHero in Liberia (a mobile phone-based communication system that connects ministries of health and health workers); Ubongo in Tanzania (that leveraged the power of entertainment, the reach of mass media, and the connectivity of mobile devices, to deliver effective, localised learning to African families at low cost and massive scale); Alerte COVID-19 in Niger (real-time alerts to solve complaints from the population on delays in screening, classifying suspicious subjects, and identifying positive cases of COVID-19) [61].

Significantly, others such as Ethiopia have used the ComBAT strategy to enhance community-based actions and testing; mSafari contact tracing in Kenya; and Integrated Laboratory Reporting System, GoData and ODK tools in Uganda, Tanzania. Additionally, others have supported other challenges linked to COVID-19, such as nutrition (for instance, Remote Integrated Phase Classification for Acute Malnutrition Analysis in Madagascar); Decentralised ART Services in Namibia; and work access permits to monitor population movement in Mauritius [61, 62].

### Resources

#### Some coordination mechanisms built on already existing coordination systems

A critical review of the coordination structure showed that the mainstay of the region’s coordination strategies to combat the COVID-19 epidemics built and strengthened the existing health systems developed during the Ebola pandemic. While enhancing the existing system was considered a crucial part of coordinating the response, it is needed to strengthen the national health system. For instance, much of Rwanda’s pandemic response adopted and leveraged existing infrastructure from Ebola preparedness efforts in 2018–19, highlighting the advantages of comprehensive pandemic preparation experience for a country [60]. The strong foundation for the initial phase of coordinating the COVID-19 response borrowed from lessons learnt during Ebola preparedness. Part of the country’s strategies, such as developing the National Preparedness Plan, training HCWs and equipping health facilities, establishing dedicated treatment centres, conducting simulation exercises, educating the public, and screening extensively at national POE, were all from Ebola preparedness.

Similarly, there were efforts to reactivate the community sensitisation structures used during the Ebola outbreak in Sierra Leone’s COVID-19 prevention phases [59]. Interestingly, officials from the MOH and Sanitation worked with diverse community leaders nationwide. While this effort was laudable, the findings show that it was not followed because it was not inclusive, as other major stakeholders – such as opposition political parties, parliamentarians, and local councillors (especially those from opposition areas) – were not adequately engaged. Nonetheless, learning from the Ebola experience, the country constituted a dedicated structure under the EOC that coordinated all stakeholders. In some countries, such as eastern DRC, where Ebola virus response was conducted in tandem with that of COVID-19, the experience showed that coordination of both the pandemics outlined the importance of different humanitarian partnerships [48].

Also, apart from the Ebola networks, other countries utilised already existing community based decentralised surveillance strategies that were built before the pandemic. For instance, in CAR, there was a pilot project using already existing networks of the community relays (teams) that was implemented in the Third district of Bangui in July in partnership with the Central African Red Cross and the Directorate General of Civil Protection [63]. The community-based surveillance teams were trained to raise awareness of COVID-19 prevention measures, detect and report suspected cases and deaths in the communities, monitor simple and moderate cases, refer serious cases to hospitals and trace contacts of infected persons. Their implementation followed the humanitarian partners who support the implementation.

#### The challenges and strengths of financing system at coordination

Financing has played a key role in the coordination of the pandemic by different countries in the region. Nearly all the countries allocated funds for the pandemic preparedness activities before they recorded the first case. These funds have been essential in supporting the coordination of the COVID related activities such as capacity building sessions, regular meetings, infrastructure upgrades, and surveillance and reporting structure. The strength of the funds in the region a multisectoral approach for sourcing the funds was taken from local governments, private organisations, and development agencies but managed by different entities to enhance transparency and accountability. Institutional financial arrangements between the government (public) and external agencies (development agencies or private partners) are an important aspect in coordination that could strengthen actions in the pandemic but could be insufficient in the absence of consultation frameworks between sectoral coordination bodies due to fragmented mobilisation of resources. External funds comprise a hefty proportion of health spending in many countries in the AFRO region and are not likely to change any time soon [64]. Also, there is evidence of a lack of proper coordination between ministries of health and finance, resulting in poor trickle-down of the funds from responsible ministry to the facilities [65].

In Mali, financing the rapid implementation of control measures for COVID-19 saw the government create a National Fund to fight COVID-19 which was done in conjunction with private sector donations. For instance, the robustness of coordination in Cameroon was managed by the United Nations in Cameroon who decided to put in place COVID-19 Basket Fund, which was designed to serve as the One COVID-19 Financing and Investment Platform [66]. To enhance accountability, the UNDP was responsible for the financial management of the Basket Fund and ensured monitoring of the implementation of the fund. Nonetheless, coordination was hampered by the complaints of corruption within the fund. Preliminary evidence in Uganda suggests that donor funding/coordination of funds was provided outside of public budgets and were not aligned with the government priorities, which may have resulted in duplication of efforts.

However, to enhance the decentralisation of coordination activities to the peripheral counties/districts, some governments such as Burkina Faso recognised the critical need to transfer funds directly to facilities and provide monetary support to frontline staff providing COVID-19 services [52]. The Government modified response directives to streamline support by centralising COVID-19 response funds from national and international partners into a single account to be disbursed to districts and facilities according to need. In Uganda, budget allocations were proposed for amendments to empower subnational authorities and frontline providers to respond to their needs on the ground [64]. However, the implementation of this kind of arrangement was sometimes haphazard, as witnessed in Burkina Faso [52]. In fact, in Kenya, the counties were slow to allocate the same funds from the National government for the COVID-19 response upon decentralising the funds to the counties. Also cited as implementation challenges were delays in the flow of funds from the national government to counties and subsequently slow flow of funds to facilities. Overall, there has been an absence of frameworks for consultations that links the sectoral coordination bodies due to fragmented mobilisation resources [52].

Equally, in Uganda, the funds were allocated based on the function. For instance, the funds meant for surveillance, sample collection, and contact tracing for districts were channelled through local governments, while those for used enforcing lockdown measures and quarantine were channelled through the Ministry of Internal Affairs, Department of Defence [64]. In Zimbabwe, the government established the National COVID-19 Response Taskforce headed by the second vice president, mobilised financial resources locally and internationally to cushion the country from the negative impacts of the pandemic [67].

### Decision

#### Roles of the heads of states and politics in the pandemic

National leadership in the response, which everyone recognises and supports by all partners, is another extremely important aspect of health emergency management. The political dynamics and leadership of different countries shape the coordination processes of the pandemic in the countries. They can either direct the coherence of the processes of management of health emergencies or disrupt them. The high-level political engagement, particularly in response to COVID-19, aligns with the global lessons learnt that take on the whole government approach [68]. For example, in nearly all the countries, the high-level political leadership and commitment led to focused efforts in coordinating response interventions. The political commitment enabled coordination response actions such as multidisciplinary engagement of health, non-health sectors (education, culture and tourism, trade and industries, transport, and law enforcement bodies), and other private partners (development partners and private companies) [69].

Specifically, some heads of states established a series of response committees and task forces as part of a multisectoral response but also doubling political figures in the countries personally lead the response. For instance, in Burkina Faso, the Prime Minister’s Cabinet established a series of response committees and task forces as part of a multisectoral response [52]; while in Botswana, the President led the Multi-Disciplinary Presidential Coordination COVID − 19 Task Team that ensured coordination of the COVID-19 preparedness and response [70]. In Chad, the Health Crisis Management Committee was created and placed under the President of the Republic’s authority and composed of eight members who oversaw the response of the pandemic [71].

However, in other countries, local politics affected coordination. For example, Sierra Leone, which was initially applauded to have an inclusive approach that encompassed political persuasions, was diluted by conflicting accusations between the government and the main opposition political party. The arrest and detention of the head of the National Ebola Response programme on allegations of attempted treason resulted in opposition party members no longer participating in the EOC [59].

### Information

#### A coordinated flow of information and joint effort in tackling misinformation

Overall, in all the countries, the government led the strategic and operational command of the response and information flow through the MOH or other governments entities. The governments’ utilised WHO guidelines to disseminate information on prevention and highlight the actions being taken by health authorities to deal with the unfolding crisis. In most cases, the official government platform provided regular updates on the COVID-19 situation through regular information broadcast, government website and social media.

Interestingly, a joint effort from the government, partners and private entities was utilised to provide digital platforms that helped combat the spread of the disease infodemic of misinformation and fear associated with COVID-19 among the communities. For example, the USAID-funded “Citizen Participation in Health Governance” project was used to restore the confidence and trust needed to manage epidemics in Guinea. Others, such as the implementing partner, Search, collaborated with Family Health International (FHI 360) to use innovative strategies of participatory theatre to dispel fear and misinformation about health centres and workers [72]. The actors portrayed real situations with themes around health and invited the audience to join in, allowing the community to be part of the solution.

## Discussion

This study has utilised the framework on the emergency response life cycle proposed by Chen et al. [23] to analyse the coordination mechanisms implemented in WHO AFRO region for the management of COVID-19. In overall, the framework – through the five elements – has been useful in thematically analysing huge set of data and information across the 47 counties of the WHO AFRO region. This study adds to the growing literature on coordination mechanisms for infectious diseases in Africa. The study reveals that the lessons from the past infectious disease crises [26–28] have been useful in changing the shape of coordination mechanisms of COVID-19 through partnerships between governments, partners, renowned experts and even the private sector agencies and which has been an essential component of reducing the response. Additionally, it has shown that a collaborative effort to collect, assemble, analyse, and make health information available to communities through coordination and collaboration at different levels of government [31] has been a useful element of the response.

Our findings have shown that different countries instituted three distinct layered coordination mechanisms (strategic, operational, and tactical) either singularly or in tandem and at varied times before or after reporting the first case and were based on the strengthen of the country systems. The organisational structures, the flow of information and tasks flow among the responders’ groups in different countries were aimed at creating scalable organisation and maintaining span of control. Consistent with our findings, some studies have shown that the layered coordination is efficient in facilitating efforts in ensuring a coordinated multisectoral readiness and early response interventions from strategic, technical and operational perspectives [69]. Besides, the layered coordination structure under unified command enhances the strategic decision making and priority setting and relied on participants that had adequate legal authority, responsibilities, and expertise across the continuum of care. Countries that had a fully layered coordination structure focused on the multisectoral systemwide resources, authorities, and expertise toward a common set of objectives while maintaining authority and direction over the jurisdiction’s health care response. The layered coordination mechanisms by different countries may have enhanced a joint integration of individual viewpoints, knowledge, and expertise of single members within the coordination network. In turn, this improved the outcome of the decision-making process, as the perception of fairness, acceptance of the decisions made, and identification of the group with decision impacts are increased and efficient in mitigating COVID-19 crises in the countries [73].

Importantly, our findings show that there is no statistically significant difference in the relationship between the type of coordination mechanism applied in the country with the length of time between onset of coordination and first case (in days); the length of wave 1; attack rate at wave 1 Peak; and attack rate at wave 2 Peak but a statistically significant difference in the coordination mechanism applied in the country with the length of wave 2. A plausible explanation could be that coordination mechanism and the level of preparedness may not strong enough in the first wave as every country was trying to do so much within a short period of time. It is more like countries were piloting to see what works and what does not work as there were many moving parts as coordination took shape. For instance, most countries did not have adequate labs to conduct testing and that a majority of the cases in the first wave were initially asymptomatic hence were not picked. Nonetheless, the broader government coordination mechanisms may have been a key to enhancing the initial wave of coordination [74].

This study has shown that coordinated flow of tasks from the central to the local government/agencies were imperative in the pandemic management. Across the countries there was effective coordination and collaboration as through inter-organisational networks [32], but varied from one country to another within the AFRO region [33, 34]. Key finding in our work was the role of heads of states and ministerial committees leading the strategic level. In particular, the high-level political leadership and commitment facilitated the efforts to ensure coordinated multisectoral readiness and early response interventions from strategic, technical and operational perspectives [69]. As has also been shown in Ethiopia, this enabled a progressive multidisciplinary engagement of non-health sectors such as education, trade and industries, transport, culture and tourism and law enforcement bodies [69]. The high political engagement aligns with global lessons learnt in response to the COVID-19 pandemic, such as the whole government approach. This finding confirms that decisive leadership was an essential ingredient in the COVID-19 response [35].

Our findings have shown that at the operational level, the countries that activated PHEOCs were responsible for early response action and mitigation measures and oversaw the preparedness and response mechanisms for any public health emergency. PHEOCs maintained situational awareness across the system at the operational level, and some mobilised and deployed local health care resources. These mechanisms were then decentralised to some countries. The clearly decentralised responses to the local regions, and each was aimed at achieving specific objectives such as the establishment of treatment centres for COVID-19 patients in all the 10 regional capitals [50] These decentralisation strategies took a synergistic approach are aimed at enhancing the operations of pandemic management, leaving the central government to focus on strategy development and resource mobilisation; but also enhance the motivation and morale of HCWs to manage COVID-19 cases. Some decentralised strategies have relied on the already existing structure used during previous emergencies such as Ebola outbreak in Sierra Leone. It became easy to reactivate the community sensitisation structures where the MOH and Sanitation officials visited and informed several community leaders nationwide about COVID-19.

The decentralisation strategies were focused on optimising the autonomous management and control of the pandemic in the different countries region’s countries for rapid and efficient care of citizens during community transmissions. In line with other literature, the decentralisation strategies enhanced the response to the pandemic; however, its success depended on the government’s political commitment to provide the required health resources/facilities [75]. This finding emphasises the vertical operation as a function of the authority to command and horizontal operations act together to achieve a common objective [20]. The different countries responses were tailored based on institutional and political contexts and it strengthened the response outcomes [30]. However, while our studies showed that some countries decentralised care from the nation level with clear lines of authorities and communication channels unlike previous research showed [31], which enhanced the uninterrupted operation of sequential tasks during emergency response, and the success of the response.

While the WHO provided the International Health Regulations (IHR) framework that sets countries to detect, monitor, report and respond to outbreaks of infectious diseases [76], our findings have shown that - despite countries in the region making progress towards achieving the IHR – the coordination mechanisms have revealed that there is still some inadequacy in pandemic preparedness, governance, leadership, and funding abound. Similar findings of the inadequacy in preparation have been shown by Looke et al. [77]; and Damme, et al. [78]. The challenges that have characterised the coordination of the COVID-19 pandemic in the African region address the heterogeneity of the roles of the stakeholders and actors involved, the diversity of activities to enhance resilience, the multi-dimension effects of different strategies, and the centrality of knowledge transfer and sharing mechanisms. This is akin to the findings by Margherita et al. [79] on managing the COVID-19 emergency. The WHO’s AFRO region Strategic Preparedness and Response Plan (SPRP), outlined the essential steps needed at global, national and local levels to suppress transmission of COVID-19, reduce exposure, protect the vulnerable and save lives [80, 81]. One of the steps was coordination, and the countries have been guided by it in their response. The SPRP 2021 updated the strategy and further guided the countries by considering new knowledge and more effective tools developed over the preceding year [82]. The WHO AFRO has developed a plan that positions the Future of WHO COVID-19 Response Operation in Africa in 2022 [83] which gives strategic orientations that are being implemented rapidly and consistently at national and regional levels to enable the continent to end the current pandemic and strengthen the foundations of a future pandemic preparedness and response architecture.

### Limitation

This study is not without limitations. For instance, we acknowledge that the countries’ variation end dates of the second wave may have biased the results. However, given that the regression focused on the length of time between onset of coordination and first case and independent peak attack rates of the different waves, we believe we overcame the bias. Also, not including other factors such as testing capacity, population density, and institution of social measures in the analysis could have biased the results. However, we believe that they likely exist in the causal pathway between coordination and epidemiology of the disease. The decisions on what to do, including public health and social measures and how testing is conducted, are informed by the coordination teams. We also excluded population density but instead used the proportion of the total population in urban areas as a better transmission indicator. There were also gaps in information in some countries, such as Burundi and Cape Verde, due to the difficulty in access. However, we relied on the FPs to provide the information based on their experience with the countries which strengthened the analysis. Other future studies could strengthen these aspects. Additionally, future researchers could relate the coordination efforts with the mortality or morbidity of COVID-19 within the WHO AFRO region.

## Conclusion

Varied coordination mechanisms are essential in building comprehensive response models through the collaboration of multiple stakeholders. Coordinating an emergency is a multidimensional process that includes having decision-makers and institutional agents define and prioritise policies and norms that contain the spread of the disease, regulate activities and behaviour and citizens, and respond to personnel who coordinate the prevention actions. Coordination mechanisms provide a structured pandemic management plan or outline of purposeful actions. Collaborative effort by different stakeholders in the COVID-19 response have collected, assembled, analysed, and made health information available to communities through coordination and collaboration at different levels of government.

## Supplementary Information

**Additional file 1.** Literature review on coordination mechanisms for COVID-19 per country.

**Additional file 2.** Demographic characteristics of the countries.

**Additional file 3.** Correlation matrix for all independent variables.

## Data Availability

Datasets used in the current study are from publicly available sources, and the extraction sheets have been added in the Additional files.

## Abbreviations

ACDC: Africa Centres for Disease Control and Prevention
COVID-19: Coronavirus Disease 2019
DP: Development Partners
GDP: Gross domestic product
IMS: Incident Management System
PPE: Personal Protective Equipment
PHEOC: Public health emergency operation centre
PSHM: Public health and social measures
RCCE: Risk Communication and Community Engagement
UN: United Nations
WHO: World Health Organization
WHO AFRO: World Health Organization Regional Office for Africa
