# Supplementary material for: Coordination mechanisms for COVID-19 in the WHO Regional office for Africa
Source: BMC Health Serv Res. 2022 May 28;22:711. doi: 10.1186/s12913-022-08035-w (PMC9142827; doi:10.1186/s12913-022-08035-w)
Supplement: Supplementary file 1 — Additional file 1. Literature review on coordination mechanisms for COVID-19 per country. [file 12913_2022_8035_MOESM1_ESM.docx]

**Additional file 1: Literature review on coordination mechanisms for COVID-19 per country**

1. **ALGERIA**

**Coordination at the national level (governance, roles, and information flow):**

- As soon as the first cases of coronavirus appeared in Wuhan, China and South Korea, the Prime Minister issued instructions to the Ministers of the Interior, Transport and Health for the coordinated implementation of an alert system at airports with flights to and from China, to be able to detect carriers of the virus and implement the usual preventive measures.
- With the spread of the virus and the declaration by the WHO of the COVID-19 pandemic as well as the first cases in Algeria, further instructions were given to the aforementioned sectors concerned in order to reinforce the preventive sanitary controls at all border checkpoints including by air, in close coordination with the competent ministry departments.
- The coordination framework comprises the following:
- Creation of a COVID-19 watch and alert cell in the Transport Ministry that monitors the spread of the virus nationally and internationally, and implementation of orders by public authorities, and;
- Creation of a multi-sector ad hoc prevention committee for monitoring and fighting the spread of COVID-19 which functions as a national crisis cell for the daily monitoring of measures implemented in the sectors and constraints and difficulties encountered^[[1]](#footnote-1)^.

**Coordination at the county/ district/ local regions (governance, roles, and information flow):**

**When did they start?**

**25/02/2020 ^[[2]](#footnote-2)^**

**Any changes/ gaps and lessons learnt?**

1. **ANGOLA**

**Coordination at the national level (governance, roles, and information flow):**

- The state of emergency started in Angola at 00.00 am on 27 March, enacted through Presidential Decree no. 81/20, of 25 March. Having been extended for three times through Presidential Decrees no. 97/20, of 9 April 2020, 120/20, of 24 April 2020 and Decree no. 128/20, of 8 May 2020, the state of emergency was in place until 11:59 pm on 25 May, having been terminated thereafter. From then on, the state of public calamity became effective, enacted by the President of the Republic through the Presidential Decree no. 142/20, of 25 May^[[3]](#footnote-3)^.
- In February, the Government of Angola had developed a National Contingency Plan to control the Coronavirus epidemic (2019-NcoV). Under the leadership of the government (Secretary of the State for Public Health), in coordination with WHO and other UN agencies, Angola Country Office (ACO) is supporting the sectoral preparedness and response plans to COVID19 focusing on: *(i) coordination; (ii)risk communication and community engagement; (iii) infection prevention and control; (iv) continuity of health care (also including nutrition); (v) continuous access to services (education and child protection); and (vi) social policy (cash transfer and social services referral)*^[[4]](#footnote-4)^
- A high-level multidisciplinary taskforce for COVID-19 response was established that instituted a multisectoral response plan involving 23 key institutions co-chaired by the state, health and interior ministers^[[5]](#footnote-5)^ Even before the first cases were detected, the Angolan government began quarantining returnees from COVID-19 affected countries^[[6]](#footnote-6)^. A swift response to protect its health system was included but not limited to: quarantine, social distancing, closure of borders with limited exceptions, suspension of schools, restaurants, and public events, and limited transportation^[[7]](#footnote-7)^. Since the first cases were detected, Angola ramped up education, testing and contact-tracing under its “National Contingency Plan to Manage the Pandemic”.^[[8]](#footnote-8)^
- Creation of multilateral partnerships in Angola’s fight against COVID-19 has been critical because its health system is under-resourced, and its economy is in a desperate state. The United Nations Children’s Fund (UNICEF), the United Nations Development Program (UNDP), the world bank, the United States Agency for International Development (USAID), and the International Monetary Fund (IMF) are some of the international partners that have assisted Angola in terms of both financial and technical support. The United States government and the European Union have both provided financial assistance to Angola to improve its testing capacity, provide training programs, enhance contact tracing and provide assistance to vulnerable populations. Cuba sent doctors and medical supplies while Qatar and Portugal provided personal protective gears. Moreover, private partner companies such as ExxonMobil, Chevron and Jack Ma Foundation have also reached out to Angola donating personal protective equipment, and funding training programs among others^[[9]](#footnote-9)^.

**Coordination at the county/ district/ local regions (governance, roles, and information flow):**

- The Ministry of Health of Angola with the support of the World Health Organization (WHO) has deployed public health experts from Luanda to other provinces to train health professionals on monitoring and preventing COVID-19^[[10]](#footnote-10)^.

**When did they start?**

- **21/03/2020 ^[[11]](#footnote-11)^**

**Any changes/ gaps and lessons learnt?**

1. **BENIN**

**Coordination at the national level (governance, roles, and information flow):**

- Sanitary cordon rather than generalized lockdown by president effective 30/03/2020^[[12]](#footnote-12)^.
- The national Covid 19 Response plan focuses on the improvement of health infrastructures and equipment, communication, provision of healthcare and capacity-building for health professionals, journalists and community members^[[13]](#footnote-13)^.
- To manage the COVID-19 outbreak, the government adopted an integrated plan early March 2020. The integrated plan is structured around five pillars: *Country-level coordination, planning, and monitoring: The country has at an early stage activated national public health emergency management mechanisms consisting of: (i) an inter-ministerial committee chaired by the Ministry of Health (MoH) and composed of core Ministries of Cooperation and Foreign affairs, Interior and Public Security, Infrastructure and Transport, and other departments that can support the response, such as Education, Social Protection, Agriculture, and Environment ; and (ii) the existing national health crisis committee also chaired by the Minister of Health and comprising the various technical teams from Ministries participating in the inter-ministerial committee, the executive secretary of the national council for the fight against malaria, tuberculosis, HIV, hepatitis and epidemics (CNLS-TP), as well as partners in the health sector*
- Centralized official government digital platform with regular updates on the covid 19 situation. Benin, the West African nation had set up a range of digital platforms to help combat both the spread of the disease and what the World Health Organization (WHO) has called “infodemic” of misinformation around it. With the help of WHO, a state-appointed team of around 15 people is using WHO guidelines to disseminate information on prevention and highlight the actions being taken by health authorities to deal with the unfolding crisis. In addition to a centralized official government platform that is freely accessible to all mobile phone users in Benin and provides frequent updates, the digital strategy team has put out several radio inserts in around 15 local languages and a range of awareness videos and press releases across different social networks. An interactive WhatsApp messaging system has also been set up and helps the new team to communicate directly with citizens both in Benin and the diaspora.

**Coordination at the county/ district/ local regions (governance, roles, and information flow):**

**When did they start?**

- First case-16/03/2020^[[14]](#footnote-14)^.
- Prevention efforts started in early February before the first^[[15]](#footnote-15)^.

**Any changes/ gaps and lessons learnt?**

- In the early phase of the pandemic, Benin, like many African countries, lacked adequate COVID-19 testing.

1. **BOTSWANA**

**Coordination at the national level (governance, roles, and information flow):**

- President declared state of emergency effective 2/4/2020
- Establishment of the Multi-Disciplinary Presidential Coordination COVID -19 Task Team, (COVID-19 PCTT) under the leadership of H.E the President of the Republic of Botswana, to ensure coordination of the COVID-19 preparedness and response. The COVID-19 PCTT will oversee and direct the work of the National COVID-19 task team chaired by the designated National Coordinator, Dr. Masupu. These two teams will be supported by Public Health Emergency Preparedness and Response Committee and specially formed multi-disciplinary sub-committees as appropriate. A secretariat will be established to support these two high level teams. The COVID-19 PCTT will oversee and provide direction, guidance, support and expert advice across the health service and the wider public service, for the overall national response to COVID-19, including national and regional and other outbreak control arrangements with a specific focus on the following three broad areas:
  - The implementation an effective “preventative” programme
  - The strengthening of the health system across the country so that it can adequately cope with testing and treating cases
  - The effective running of the economy to ensure a positive growth rate^[[16]](#footnote-16)^.

**Coordination at the county/ district/ local regions (governance, roles, and information flow):**

- With this assistance, USAID is building upon existing community health platforms that have been strengthened over many years through PEPFAR investments to combat the HIV epidemic in Botswana.  USAID is assisting District Health Management Teams to mobilize community health workers to conduct surveillance, detection, and contact tracing during the pandemic to keep people safe.  USAID is also strengthening the healthcare system by working with local organizations and local community members who know best what the communities need.
- With timely outreach information that includes radio messages and social media posts, USAID ensures that citizens in rural and urban areas are well-informed about the risks of COVID-19 and know how to protect themselves and their loved ones.  USAID is helping the GoB reach people living with HIV and other underlying health conditions who may face greater vulnerabilities to COVID-19.  Successful risk communication decreases transmission, combats misinformation, and saves lives.
- Finally, USAID is supporting Botswana’s Ministry of Health and Wellness to establish an emergency supply chain structure so the people of Botswana can access life-saving medication, even during times of extreme social distancing and interrupted supply chains throughout the world ^[[17]](#footnote-17)^.
- With technical and financial support from UNICEF, Botswana’s Ministry of Health and Welfare (MoHW) has managed to consistently provide up to date data for decision making by scaling up the use of a COVID-19 Surveillance and Response Tracker in DHIS2 ^[[18]](#footnote-18)^.

**When did they start?**

- First case 30/03/2020^[[19]](#footnote-19)^

**Any changes/ gaps and lessons learnt?**

1. **BURKINA FASO**

**Coordination at the national level (governance, roles, and information flow):**

- The Prime Minister’s Cabinet established a series of response committees and task forces as part of a multisectoral response
- The COVID-19 Ministerial Management Committee has developed an official COVID-19 response plan, which guides the activities of the thematic commissions.
- Since the initial outbreak, the Government has modified response directives to streamline support between June and July 2020: *Centralized COVID-19 response funds from national and international partners into a single account by the Central Government to be disbursed to districts and facilities according to need.*


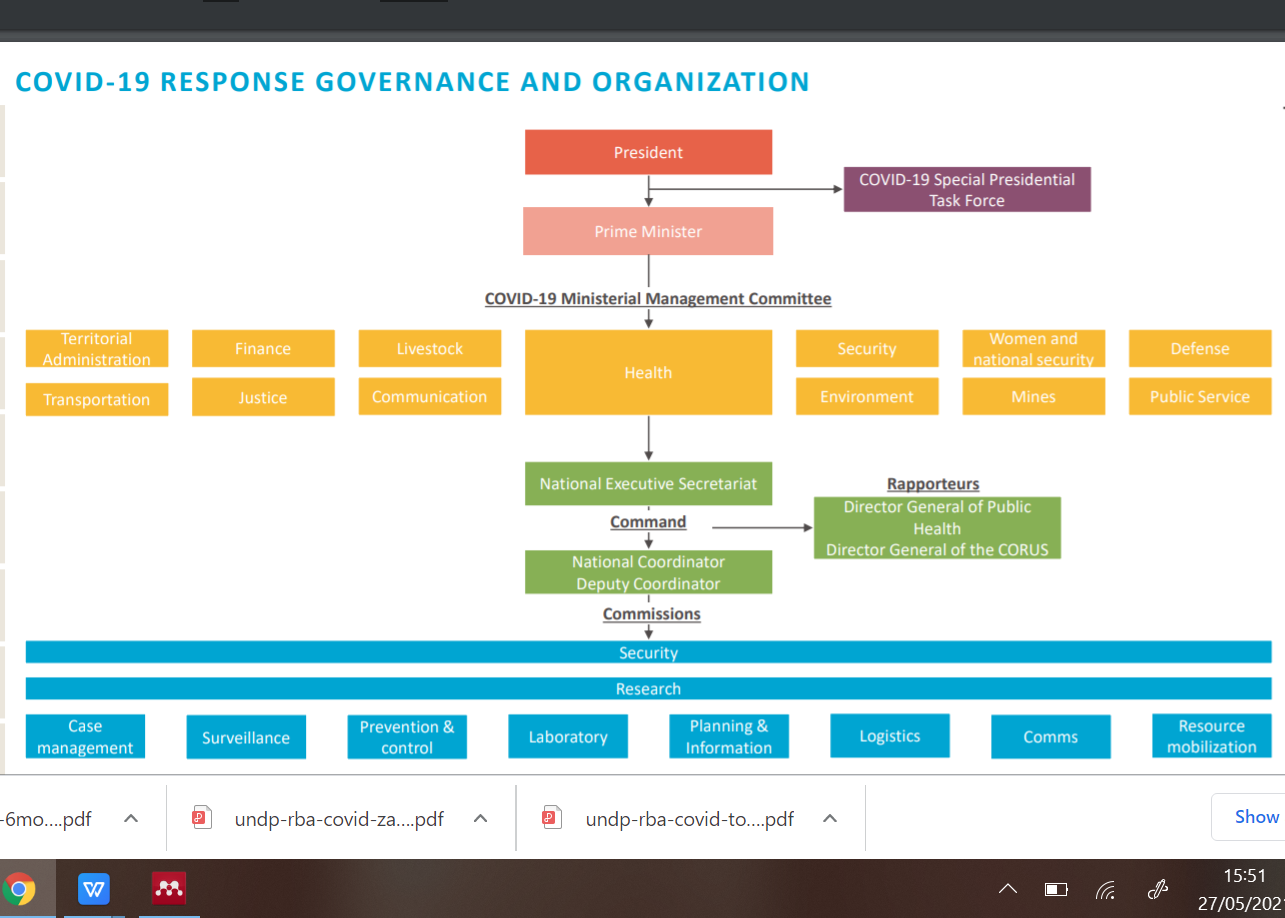


**Coordination at the county/ district/ local regions (governance, roles, and information flow):**

- Decentralized COVID-19 testing (rapid diagnostic tests) to district hospitals (CMA); Decentralized coordination of commodities and PPE to the regional level; Decentralized treatment of COVID-19 to all health facilities in the health system ^[[20]](#footnote-20)^

**When did they start?**

- 2/3/2020**^[[21]](#footnote-21)^**

**Any changes/ gaps and lessons learnt?**

- Coordination management of COVID-19 is insufficient; there is an absence of consultation frameworks between sectoral coordination bodies due to fragmented mobilization resources.

1. **BURUNDI**

**Coordination at the national level (governance, roles, and information flow):**

**Coordination at the county/ district/ local regions (governance, roles, and information flow):**

**When did they start?**

- **31/03/2020**

**Any changes/ gaps and lessons learnt?**

1. **CABE VERDO**

**Coordination at the national level (governance, roles, and information flow):**

**Coordination at the county/ district/ local regions (governance, roles, and information flow):**

**When did they start?**

- **19/03/2020**

**Any changes/ gaps and lessons learnt?**

1. **CAMEROON**

**Coordination at the national level (governance, roles, and information flow):**

- The Government prepared a COVID-19 Preparedness and Response Plan.
- The health response strategy under the leadership of the Ministry of Public Health has eight components: *i) Multisectoral and international coordination, ii) Surveillance for early detection of cases, iii) Investigation and rapid intervention teams, iv) Laboratory capacities, v) Infection prevention and control measures in hospitals and in the community, vi), Cases management, vii) Risk communication and Community engagement, and viii) logistics.*
- UNDP is partnering with the Ministry of Finance, Ministry of Economy, Planning and Regional Development on socio-economic impact assessment and response. A socioeconomic response plan is being developed with the support of UNDP. This plan is based on five pillars: *(i) strengthening the health system, ‘ii) social resilience, (iii) macroeconomic and financial stability, (iv) strategic supply, (v) research and innovation.*
- The United Nations in Cameroon has decided to put in place COVID 19 Basket Fund which has been designed to serve as the One COVID-19 Financing and Investment Platform. UNDP is responsible for the financial management of the Basket Fund and ensures monitoring of the implementation of the Fund.
- UNDP Cameroon and WFP are co-leads on Operations and Logistics support in the UN Response Plan and partnering with the World Bank and the Global Fund on medical supplies.

**Coordination at the county/ district/ local regions (governance, roles, and information flow):**

**When did they start?**

- 5/03/2020^^[[22]](#footnote-22)^^

**Any changes/ gaps and lessons learnt?^[[23]](#footnote-23)^**

- Adaptation and collaboration are key
- Instilling behaviour change in the short-term is difficult
- Early on lack of state and non-state coordination

1. **CENTRAL AFRICAN REPUBLIC**

**Coordination at the national level (governance, roles, and information flow):**

**Coordination at the county/ district/ local regions (governance, roles, and information flow):**

- As part of its decentralization strategy, the Ministry of Health has introduced a community-based surveillance strategy. A pilot project was implemented in the 3rd district of Bangui in July in partnership with the Central African Red Cross and the Directorate General of Civil Protection. The community-based surveillance teams are trained to raise awareness of COVID-19 prevention measures, detect and report suspicious cases and deaths in the communities, monitor simple and moderate cases, refer serious cases to hospitals and trace contacts of infected persons. The implementation of community-based surveillance in the rest of the country will follow. Humanitarian partners will support the implementation through existing networks of the community relays^[[24]](#footnote-24)^.

**When did they start?**

- 14/03/2020

**Any changes/ gaps and lessons learnt?**

- **Fragile health system:** First, the health system is barely functioning, due to a chronic shortage of skilled health workers, medical equipment and basic medicines. Seventy per cent of health services are provided by humanitarian organizations and over 2.5 million people, half of the population, need health assistance. One in four Central Africans needs to walk for over an hour to reach the nearest clinic and for many, the bills for consultations and medications are unaffordable. When it comes to one of the most basic measures to avoid contracting COVID-19 – regular hand-washing with soap and water – the situation does not look brighter. Only one in three Central Africans has access to clean water, a toilet and shower. And for many, soap is a luxury good. Access to water and sanitation is particularly problematic at the many sites where 235,000 internally displaced people (IDP) live, often in crowded makeshift shelters where physical distancing is not practicable.
- **Supplies and logistics***:* The provision of protective equipment and medical devices to diagnose and treat COVID-19 patients posed a serious challenge, particularly at the onset of the pandemic. Six months into the pandemic, the country possessed only [two ventilators](https://reliefweb.int/sites/reliefweb.int/files/resources/interagency_covid19_dashboard_good_monitoring_31_august_2020_en_a4_vf.pdf) and 25 in the pipeline, representing three per cent of the identified needs. Poor road infrastructure and the six-month rainy season disrupt supply chains to large parts of the country. Insecurity further hinders access to services and the possibility of humanitarians to reach people in need of assistance. A severe lack in cold chain infrastructure further impedes the safe supply of medicines across the country.
- **Humanitarian partners** established a tracking initiative to monitor misinformation and fear associated with COVID-19 among the communities. Over 1,300 community feedbacks were gathered and analyzed to adapt the response and communication efforts. Health and protection actors developed and disseminated key messages on stigmatization, misinformation and manipulation related to COVID-19, for example in the [Weekly Bulletin on COVID-19 rumors and Information](https://reliefweb.int/report/central-african-republic/bulletin-hebdomadaire-de-v-rification-des-rumeurs-et-informations-2). Over 10,000 radios were distributed between April and July 2020 alone and 90 per cent of Central Africans were reached with lifesaving information on COVID-19^[[25]](#footnote-25)^

1. **CHAD**

**Coordination at the national level (governance, roles, and information flow):**

- Faced with the magnitude of the situation, the Chadian authorities announced on 24th March, 2020, the establishment of a Health Surveillance and Security Committee as well as a 15 billion CFAF (approx. USD 25 million) contingency plan for the fight against the coronavirus.
  Among the measures taken by the Committee were the establishment of a coordination platform and a care facility for suspected or confirmed cases, quarantine measures for persons from countries at risk, the suspension of flights to N’Djamena beginning from 18th March, and the establishment of an emergency response team.
- Health Measures**:** Setting up of a National Coordination of Health Response led by scientists and dedicated to the operational fight against COVID-19 (CNRS-COVID-19), Inclusion of deep Chad in the health response strategy with the establishment of the Provincial Health Response Coordination (CPRS), led by eminent specialists in each province, Creation of five (5) specialized inclusive sub-committees dedicated respectively to sensitization, assistance to the poor, defence and security^^[[26]](#footnote-26)^^.
- On 16 May, a Health Crisis Management Committee (Comité de gestion de la Crise Sanitaire, CGCS) was created and placed under the authority of the President of the Republic, Idriss Déby. Composed of eight members, the Committee is in charge of leading the fight against COVID-19, in particular through the establishment of a national health response coordination led by scientists and dedicated to the fight against COVID-19, the creation of five specialized, inclusive sub-committees (on awareness raising, finance and orders, assistance to the poor, resource mobilization, and defense and security), the urgent request for essential medical equipment supplies, the acquisition of at least five mobile laboratories, the implementation of a strengthened action plan to support the 2020 agricultural campaign, the launch of food distribution operations for the poor, and the now-free distribution of masks.
- Under the leadership of the World Health Organisation (WHO) in supporting the Government’s efforts and coordinating the overall health response to COVID-19, United Nations agencies and Non-Governmental Organizations (NGOs) have started to implement various activities and initiatives on the prevention and response to the pandemic, in accordance with the updated 2020 Humanitarian Response Plan (HRP) and the contingency plan prepared by the Government of Chad. For example, awareness-raising initiatives for the population both in the capital and in the provinces - including IDP sites and refugee camps - direct awareness-raising campaigns for health representatives, training of trainers for health personnel on infection prevention and control (IPC) / WASH, as well as IPC / WASH assessments of health facilities in the capital and in the provinces. Efforts have also been made to ensure that official communications on prevention from the Ministry of Public Health are translated into local languages and disseminated through various community outreach channels, including radio^^[[27]](#footnote-27)^^.

**Coordination at the county/ district/ local regions (governance, roles, and information flow):**

**When did they start?**

- 19/03/2020^^[[28]](#footnote-28)^^

**Any changes/ gaps and lessons learnt?**

- The Government is supporting the continuation of humanitarian action during this crisis. Current preventive measures do not, in principle, affect the transport of humanitarian goods. However, as restrictions on movement are tightened, it has become more difficult to ensure humanitarian access in some areas. Certain activities have been suspended due to the stricter application of confinement measures in certain provinces, which has made it difficult for humanitarian personnel to move outside of urban centres, including N'Djamena.
- In order to ensure the essential running of humanitarian actors’ operations in the field, the Government has granted special authorizations allowing the circulation of United Nations agencies and international NGOs to support critical programmes in each province: health emergency, food distribution, WASH and shelter programmes, and vaccination campaigns. Humanitarian actors ensure all necessary precautions are taken and follow appropriate standard operating procedures during their movements and programme implementation, in order to reduce any potential transmission of COVID-19 through humanitarian action itself.
- Although the emergency response to COVID-19 warrants special attention, the existing challenges faced by the Chadian population should not be neglected. The rainy season has begun and already caused some population displacements following floods and destruction of shelters. Food insecurity is expected to worsen during the upcoming lean season. The Lac province is experiencing an increase in criminal activity by non-state armed groups, which could result in new population movements in the province and across the borders. The measles epidemic remains a major concern, since 1 January 2020, 8,026 cases and 37 deaths have been reported: 28 districts (out of 126) have been confirmed to be in an epidemic situation since the beginning of the year^[[29]](#footnote-29)^.

1. **COMOROS**

**Coordination at the national level (governance, roles, and information flow):**

- H.E. President Azali Assoumani gave a televised address to the population on March 16th and announced the end of public ceremonies such as weddings and commemorations. Government of the Comoros created a Permanent Executive Committee on March, 18th for the coordination of the preparedness and response on such topics as logistics, funds raising and management, healthcare, data collection and analysis^[[30]](#footnote-30)^
- On April 3, 2020, the National COVID 19 Pandemic Coordination Committee was established by Presidential Decree. The Committee was structured with sub-committees on logistics, communication, security, economy support, administrative and financial as well as scientific matters. A national plan was developed and adopted by the Presidency of the Republic to serve as a framework and working tool for the National Committee. The plan was to reinforce the preparation capabilities, including an alert and response system to a possible outbreak with several planned scenarios^[[31]](#footnote-31)^.

**Coordination at the county/ district/ local regions (governance, roles, and information flow):**

**When did they start?**

- 30/04/2020^[[32]](#footnote-32)^

**Any changes/ gaps and lessons learnt?**

1. **CONGO**

**Coordination at the national level (governance, roles, and information flow):**

- The **COVID-19 Technical Secretariat** and **Presidential COVID-19 Task Force** will help to guide the health system’s response *and* inform broader political decisions.
- The COVID-19 Technical Secretariat Led by Dr. Jean-Jacques Muyembe, the new **COVID-19 Technical Secretariat** will direct the health system’s response to the pandemic. Dr. Muyembe has been given autonomy over scientific *an*d public health decisions—emphasizing the government’s belief that the response should be based in science above all else.
- The secretariat will run in parallel to the Ministry of Health, allowing the ministry to focus on the country’s routine health needs and other ongoing outbreaks (measles, and the final vestiges of Ebola). This approach will help protect the country’s citizens from two looming threats: COVID-19 and potential shortages of health resources.
- Building off the lessons and capacity of the [Emergency Operations Center](https://www.path.org/articles/digital-congo-ebola/), this response will continue to build the country’s capacity and reputation as a leader in outbreak management in Africa.
- The Presidential COVID-19 Task Force will coordinate cross-sector decisions for the government at both the national and provincial levels. Led by Dr. Roger Kamba and a team of experts, this task force provides President Tshisekedi a direct line of oversight into the COVID-19 outbreak and the response coordination^[[33]](#footnote-33)^.
- During the first months of the response, all coordination meetings were carried out remotely. In eastern DRC, the memory of Ebola (and what became known as “Ebola business”) meant that it was particularly complicated for aid organisations when the Covid-19 virus arrived. As analysed in the research paper, “Observing COVID-19 in Africa through a Public Authorities Lens”7 and as reported by some interviewees, local (state and provincial) authorities have been accused of embezzling money earmarked for the response to the pandemic. Covid-19 is seen as the latest “business” for local public authorities and internationals. In this context, the legacies of conflict, Ebola and the relatively low incidence of Covid-19 has combined with a widespread lack of trust in the state and the actions of public authorities to undermine belief in the virus’ very existence. These emerging patterns highlight the importance of understanding the image of international actors as well as the role of public authorities (not just the state) in terms of the Covid-19 response. They also highlight the importance of previous experiences and the memory of such experiences in the acceptance of responses to future crisis situations^[[34]](#footnote-34)^
- Professor Jean-Jacques Muyembe, the eminent Congolese virologist who discovered the Ebola virus in 1976 and who is leading the national response to Ebola and COVID-19.

**Coordination at the county/ district/ local regions (governance, roles, and information flow):**

**When did they start?**

- **10/03/2020**

**Any changes/ gaps and lessons learnt?**

- Most of Africa’s health systems are extremely fragile, and epidemics such as Ebola and COVID-19 only underscore this fact. Our previous experiences have shown us that if we hope to succeed, the mainstay of our strategies to combat epidemics in general and Ebola in particular must be the existing health systems, which should be strengthened when epidemics surface. This is critical, and it must not be allowed to weaken the national health system
- National leadership in the response, which is recognized by everyone and supported by all partners, is another extremely important aspect of health emergency management.
- The Ebola virus response in eastern DRC was a prime example of the importance of partnerships^[[35]](#footnote-35)^.

1. **COTE D’IVOIRE**

**Coordination at the national level (governance, roles, and information flow):**

- The Government has designed a national emergency response plan and has just established the Public Health Emergency Operations Center (COUSP). A crisis committee to better manage the risk of the disease spreading has been putted in place^[[36]](#footnote-36)^.
- The government of Côte d’Ivoire created an operational monitoring committee for the fight against COVID-19. The committee is made up of nine sectoral operational coordination units and key actors. Working with USAID technical implementing partners, the committee organizes and synthesizes a range of essential community services, including testing, containment and epidemiological surveillance, medical care, hygiene, health security, burials, and pharmaceutical stock management and distribution.
- The Government has established an organizational framework for coordination, prevention and control of the coronavirus COVID-19. The framework is composed of a National Steering Committee, chaired by the Prime Minister and the MSPH as secretariat, and several subcommittees, and action units in each sector. The Steering Committee provides both strategic and policy guidance and oversight for the Governments' emergency response; it coordinates the activities of the other committees and mobilizes resources for the implementation of the Government's emergency response plan and response. The secretariat of the Steering Committee Is led by the MSPH.
- At the operational level, the MHPH's main body for multisectoral coordination to public health emergencies responses called the Centre for Public Health Emergency Operations (COUSP) chaired by INHP has been activated. All health key stakeholders including the World Bank and WHO are invited to participate in the weekly coordination meeting organized by COUSP. The operational response of COVID-19 is led by the General Director of Health under the leadership of the MOH.

**Coordination at the county/ district/ local regions (governance, roles, and information flow):**

**When did they start?**

- **12/03/2020**

**Any changes/ gaps and lessons learnt?**

- Despite these efforts, the emergency preparedness capacity of the country still requires strengthening. CIV urgently needs to accelerate response planning. Its initial Emergency Preparedness and Response Plan assumed COVID-19 cases would arrive from China. But as the country is now already affected by the disease, the preparedness and response plan has been revised to assume community transmission^[[37]](#footnote-37)^
- Throughout this crisis, the health system has been experiencing significant challenges, especially in terms of coordination, and the inadequacies of various sectors of the health system are being exposed^[[38]](#footnote-38)^.
- The support provided by HRH2030 will be useful in three keyways: harmonizing management procedures between laboratories; promoting teamwork and the implementation of the One Health platform with effective collaboration for the management of future infections; and strengthening the role of the committee to synchronize field interventions and increase the availability of data to inform decision-making.

1. **EQUITORIAL GUINEA**

**Coordination at the national level (governance, roles, and information flow):**

- The government, in collaboration with WHO and other partners, has developed an Emergency Plan for Prevention and Contingency for possible cases of coronavirus and a broader response plan with a whole of government approach.^[[39]](#footnote-39)^.
- On March 20, 2020, the President issued a decree (the “March 20 Decree”) to mobilize economic and material resources against coronavirus^[[40]](#footnote-40)^.

**Coordination at the county/ district/ local regions (governance, roles, and information flow):**

**When did they start?**

- **14/03/2020**^[[41]](#footnote-41)^

**Any changes/ gaps and lessons learnt?**

1. **ERITREA**

**Coordination at the national level (governance, roles, and information flow):**

- Prior to the first confirmed case, a national taskforce was established, comprising government high-level officials, health authorities, and experts, to lead the country’s response to the pandemic. In collaboration with UNICEF, the WHO, and other international organizations, a National Preparedness and Response Plan was developed, and proactive steps were taken to strengthen the healthcare system. On March 11, ten days before the country’s first confirmed positive case, the government released a statement outlining the global dimensions of COVID-19
- Local factories, in collaboration with the MoH, shifted production to manufacture hand sanitizer, soaps, and facemasks, and these products were quickly distributed to health facilities, offices and ministries, schools, and vulnerable populations across the country
- Eritrea’s response has been characterized by clear, consistent, and frequent communication, as well as local buy-in and adherence
- with Eritrea being an ethnically diverse, multilingual country, public service messages and information about COVID-19 were developed and translated into all nine local languages (as well as sign language) and regularly disseminated through various forms of media (national television and radio)^[[42]](#footnote-42)^.

**Coordination at the county/ district/ local regions (governance, roles, and information flow):**

**When did they start?**

- 21/03/2020^[[43]](#footnote-43)^

**Any changes/ gaps and lessons learnt?**

1. **ESWATINI**

**Coordination at the national level (governance, roles, and information flow):**

- Following the command by His Majesty King Mswati III and Ingwenyama to declare the outbreak of coronavirus as a National Emergency, Government has extensively consulted to roll out the relevant structures that will implement the National Emergency Plan. These include a National Emergency Management Committee (Cabinet sub-committee) that will be chaired by the Deputy Prime Minister and a National Emergency Task team that will operationalise the implementation of the Response plans.
- The Members of the National Emergency Management Committee are tasked with the following responsibilities, among others:
- Coordinate National and Regional Emergency management measures at Cabinet level
- Provide Cabinet oversight of the National Emergency Management Task Force
- Act as the interface between the Government and the international community regarding emergency management and assistance matters.
- Government has furthermore appointed a National Emergency Task Force, comprised of members of different sectors of society to implement National and Regional Emergency Plans and Procedures, and Coordinate preparedness and response activities. Other duties of the Task Team include the following:
- Establishing and directing the policy of the National Emergency Management Secretariat with respect to planning, organizing, equipment, training, administration and operations.
- Enlisting suitable persons to be volunteers in the coronavirus response.
- Inspecting at regular intervals, resources for preparedness and response activity.
- Mount multi-organizational exercises as may be necessary to test emergency plans and procedures^[[44]](#footnote-44)^

**Coordination at the county/ district/ local regions (governance, roles, and information flow):**

- There will be Regional Committees to be chaired by Regional Administrators and various Sector Committees that will coordinate the response and implementation plan.
- The Deputy Prime Minister responsible for chairing the Task Force ensuring it implements emergency plans throughout all the regions of the Kingdom to reach every community^[[45]](#footnote-45)^.
- Elaborate stakeholder engagement plan-state and non-state^[[46]](#footnote-46)^

**When did they start?**

- **First case-13/03/2020**^[[47]](#footnote-47)^

**Any changes/ gaps and lessons learnt?**

1. **ETHIOPIA**

**Coordination at the national level (governance, roles, and information flow):**

- The Government has strengthened its preparedness and response efforts to combat COVID-19 and has set up a well-organized national preparedness and response coordination mechanism through an Emergency Operation Centre.
- Ethiopian government has set up four different levels of coordination: *(a) National Disaster Risk Management Council led by the deputy prime minister’s office; (b) Public Health Emergency Management (PHEM) incorporated a multi-sectorial national task force led by the Minister of Health; (c) The PHEM technical taskforce that has been managed by the Director-General of Ethiopian Public Health Institute (EPHI); and (d) PHEM Technical Working Group led by the national incident manager.*
- As of 31^st^ March 2020, a synergistic approach COVID-19 humanitarian actions have been coordinated by the established Emergency Coordination Centre and national and regional the task forces were established in all regions^[[48]](#footnote-48)^.
- **COVID-19 Guideline and Protocol Development-** Ethiopian government developed standards, guides and protocols documents to respond to COVID-19. The documents have been developed by assessing the situations, impacts and risks of COVID-19 pandemic. Important documents *a) risk communication and community engagement mechanism in Ethiopia* that aimed to have and create a mutual understanding of preparedness and response to minimize the effects of COVID-19; *b) cleaning and disinfection Protocol for COVID −19* which helps to communicate and inform respected stakeholders in well-designed manner. *c) Some other document on Project Information Document could be developed by the World Bank.* The aim of the World Bank Project is to make a holistic approach to the impact posed by COVID-19. Besides, the project expected to strengthen national systems or taskforces for its preparedness in Ethiopia. *d) National Implementation Guide for COVID-19 Home-Based Isolation and Care* and its objectives of the guide are to outline the steps and the procedures; describe the roles and responses; and provide detailed technical and administrative guidance. *e) Quarantine and Border Control Implementation Guide* considering the increased risk impact of imported cases to the country was developed^[[49]](#footnote-49)^.

| Protocol | health care facility COVID-19 Preparedness and response | MoH & EPHI | - |
| --- | --- | --- | --- |
| Protocol | Case management | MoH & EPHI | March 2020 |
| Protocol | Cleaning and disinfection, routine environmental cleaning | MoH & EPHI | June 2020 |
| Protocol | Screening and Quarantine of Cross-Country Truck Drivers and Other Crews | MoH & EPHI | - |
| Plan | National Action Plan | MoH & EPHI | - |
| Strategy | RCCE for Ethiopia | MoH & EPHI | 2020 |
| Project Information Document | Ethiopia COVID-19 Emergency Response | WB | March 2020 |
| National Implementation Guide | Home-Based Isolation and Care | MoH & EPHI |  |

**Coordination at the county/ district/ local regions (governance, roles, and information flow):**

- Regional COVID-19 Coordination Arrangements

| **Region** | **Motivated** | **Stakeholders** | **Leaders** |
| --- | --- | --- | --- |
| Amhara National Regional State | Emergency Committee | Regional office | Regional head of EPHI |
| Afar National Regional State | Task Force | Regional office | Regional President |
| Benishangul Gumuz National Regional State | Steering Committee | Cabinet members | Regional President |
| Gambela National Regional State | No formal Forum | Health cluster members | Regional Health office/WHO co-chairs |
| Oromia National Regional State | Steering Committee | Regional office heads and partners | Office of the President & ODRMC |
| SNNP | Task Force | Regional office heads | Regional President |
| Somali National Regional State | Regional State EOC | Regional office heads and partners | Regional HB |
| Tigray National Regional State | Regional State EOC | Regional office heads and partners | Regional Health office |
| Harari | Task Force | Regional office heads and partners | Regional President |
| Dire Dawa | Steering Committee | City office heads | City Mayor |

- ***Source:*** *Data from: NDRMC. ETHIOPIA: COVID-19. Humanitarian impact Situation Update No. 01 National Emergency Coordination Center. 2020^[[50]](#footnote-50)^*

**When did they start?**

- **13/03/2020^[[51]](#footnote-51)^**

**Any changes/ gaps and lessons learnt?**

1. **GABON**

**Coordination at the national level (governance, roles, and information flow):**

- In terms of Government response mechanisms, the central level now has (Presidential and Prime Minister’s level) a coordination unit, but there is no provincial-level coordination mechanism^[[52]](#footnote-52)^.
- A National Steering Committee was set up under the supervision of the Prime Minister, as well as a national response committee coordinated by the Ministry of Health and the Military Health Services.
- In order to strengthen the response, the country has set up a scientific committee whose role is to provide scientific and technical support to the steering committee. The steering committee, with support from WHO, updated the COVID-19 epidemic response plan, currently budgeted at 19.5 billion francs CFA (equivalent to US$32.2 million) The response plan covers 6 main areas: epidemiological surveillance including Points of Entry; case investigation and solation (including contact tracing); risk communication and community engagement; infection prevention and control (incorporating WASH); laboratory diagnostics; clinical case management; and logistical and administrative support.
- The UN system through WHO is providing extensive technical assistance (TA) in terms of the coordination, risk communication and community engagement, surveillance and Rapid Response Teams (RRTs), and Points of Entry. UNICEF, UNESCO and UNDP are the main lead agencies for social protection interventions.

**Coordination at the county/ district/ local regions (governance, roles, and information flow):**

**When did they start?**

- **13/03/2020^[[53]](#footnote-53)^**

**Any changes/ gaps and lessons learnt?**

1. **GAMBIA**

**Coordination at the national level (governance, roles, and information flow):**

- The Gambia established a PHEOC in 2016 following the Ebola epidemic, recognizing its value-add to the country’s public health emergency management system. The Gambian Covid-19 response has been steered by the multi-hazard emergencies operations agency (the National Disaster Management Agency – NDMA), under which the Gambian PHEOC has managed the public health dimensions of the response.
- While in its early stages of development, the Gambian PHEOC has played a central role in coordinating the response to Covid-19, including conducting risk evaluation and diagnosis, response preparation and planning, surveillance, and monitoring^[[54]](#footnote-54)^.

**Coordination at the county/ district/ local regions (governance, roles, and information flow):**

**When did they start?**

- **17/03/2020^[[55]](#footnote-55)^**

**Any changes/ gaps and lessons learnt?**

- The Gambian Public Health Emergency Operation Centre’s operational limitations have been offset by unsustainable levels of personal commitment from the Ministry of Health personnel, which threatens the efficiency of the response, particularly for a crisis managed over an extended period of time rather than the short-term^[[56]](#footnote-56)^.

1. **GHANA**

**Coordination at the national level (governance, roles, and information flow):**

- With the nature of Ghana’s government, public policy mainly emanates from the Executive President at the center which is then translated to the regional and local government levels whose heads are appointees of the President for implementation. It is a result of this that the President adopted a televised address to the nation to update citizens on the measures put in place by the government as a response to the crisis. In these addresses are where all policies were outlined for various Ministries, Department, and Agencies to follow up with implementation. Critical to the fight has been the earlier admission by the President on the potential of the disease to wreak havoc if proper care is not taken.
- The early admission is evident in the President’s first address on “the enhanced measures taken by the government” on 11^th^ March 2020 when the country had not reported any case of COVID-19. In this address, the President outlined some strategies the country was adopting to even prevent the COVID-19 from entering the country.
- An Inter-Ministerial Presidential taskforce on COVID-19 was constituted with the President at the head and chairing most of the meetings. This brings to fore the importance attached to the emergency responsiveness and coordinated measures outlined in the public policy responses^[[57]](#footnote-57)^.

**Coordination at the county/ district/ local regions (governance, roles, and information flow):**

**When did they start?**

- **12/03/2020**

**Any changes/ gaps and lessons learnt?**

1. **GUINEA**

**Coordination at the national level (governance, roles, and information flow):**

- [Well before the first cases of COVID-19 surfaced in Guinea, the country had devised a response strategy](https://twitter.com/intent/tweet?text=well+before+the+first+cases+of+COVID-19+surfaced+in+Guinea%2C+the+country+had+devised+a+response+strategy&url=https://blogs.worldbank.org/nasikiliza/guinea-knowledge-comes-experience-how-lessons-learned-combating-ebola-led-quick-response/?cid=SHR_BlogSiteTweetable_EN_EXT&via=WorldBankAfrica), had established a budget for which funding had already been mobilized, and was equipped with knowledge gained from combating Ebola ^[[58]](#footnote-58)^.
- In order to contain the virus, limit its spread and break the transmission chains, the government, through the National Agency for Health Security (ANSS), has put in place several mechanisms and activities which are, in part, drawn from 2014 Ebola epidemic.
- *Coordination* (Frequent concertation meetings between the ANSS and relevant stakeholders to monitor the sanitary situation; Establishment of the Center for Operations of Public Health Emergency (COU-SP) in alert mode).
- *Surveillance* (Surveillance measures at strategic entry points have been strengthened. Establishment of identification tools and Standard Operating Procedures. Deployment of contact tracing teams in the capital and establishment of a COVID-19 Hotline).
- *Patient Care* (03 fully operational treatment centers (TCs) have been established in Conakry, Boké and Kindia. Provision of medicine and inputs for patient treatment to all 03 TCs. Patient transfers from entry points to TCs in less than 1 hour now effective).
- *Communication* (Regular information broadcast on COVID-19 on the ANSS website and social media. Regular broadcast on best practices by telecom operators as well as TV and radio).
- *Logistics* (Provision of medicine and equipment for patient treatment to all 03 TCs)^[[59]](#footnote-59)^.

**Coordination at the county/ district/ local regions (governance, roles, and information flow):**

**When did they start?**

- **13/03/2020^[[60]](#footnote-60)^**

**Any changes/ gaps and lessons learnt?**

- Before COVID-19, there was Ebola — a virus that ravaged West Africa and fueled fear that tore at the social fabric of communities. The stories and lessons learned from Ebola have never been so relevant. In 2013, the Ebola virus hit Guinea. The lack of trust between communities, health workers, and authorities hindered its eradication. In two years, 2,543 citizens died. Afterward, many people saw health centers as hotbeds for Ebola. This fear dissauded people from receiving vaccinations and other needed health services.
- Since 2017, the USAID-funded "Citizen Participation in Health Governance" project has been restoring the confidence and trust needed to manage epidemics in Guinea. As an implementing partner, Search collaborates with Family Health International (FHI 360) to use participatory theater to dispel fear and misinformation about health centers and workers. Actors portray real situations with themes around health and invite the audience to join in, allowing the community to be part of the solution^[[61]](#footnote-61)^.

1. **GUINEA BISSAU**

**Coordination at the national level (governance, roles, and information flow):**

- The Government of Guinea-Bissau has developed a National COVID-19 Contingency Plan. The Plan focuses on scaling-up and strengthening all aspects of preparedness and response including emergency coordination, health vigilance, communication and social mobilization, prevention and infection control and clinical management^[[62]](#footnote-62)^.

**Coordination at the county/ district/ local regions (governance, roles, and information flow):**

**When did they start?**

- **25/03/2020^[[63]](#footnote-63)^.**

**Any changes/ gaps and lessons learnt?**

1. **KENYA**

**Coordination at the national level (governance, roles, and information flow):**

- The Ministry of Health (MOH) issued the National 2019 Novel Coronavirus Contingency (Readiness and Early Response)
- February 2020: President Kenyatta issued an Executive Order on February 28 to establish the *National Emergency Response Committee (NERC)* on Coronavirus
- NERC responsibilities: *1) Coordinate Kenya’s preparedness and response to COVID-19 2) Coordinate building capacity of medical personnel and other professionals 3) Enhance surveillance at all points of entry 4) Coordinate the preparation of national, county, and private isolation and treatment facilities 5) Coordinate the supply of testing kits, critical medical supplies, and equipment 6) Conduct economic impact assessment and developing mitigation strategies 7) Coordinate technical, financial, and human resources efforts with development partners and key local stakeholders 8) Formulate, enforce, and review processes and requirements that regulate entry of people travelling from COVID-19 affected countries*
- *National covid-19 task force leads implementation of contingency plan*.
- Roles – Convene COVID-19 Task Force (chaired by the Principal Secretary) and outbreak coordination meetings; Prepare and release daily and weekly situation reports; Prepare regular media updates; Conduct regular risk and needs assessment; Manage communication hotlines; Facilitate simulation exercises

**Coordination at the county/ district/ local regions (governance, roles, and information flow):**

- May 2020 — The Council of Governors (COG) established the cross - sector COVID -19 Secretariat to coordinate counties’ response and recovery strategy

**When did they start?**

- Response began before cases-January 2020: The Ministry of Health (MOH) issued the National 2019 Novel Coronavirus Contingency (Readiness and Early Response)
- First case- 13/03/2020

**Any changes/ gaps and lessons learnt?**

- Challenges: counties were slow to allocate funds for the covid-19 response, delays in flow of funds from national government to counties, slow flow funds to facilities, Potential of NHIF to purchase services from private facilities and re-enforce medical cover for front-line health workers is largely untapped.

(All info is from Thinkwell^[[64]](#footnote-64)^)

1. **LESOTHO**

**Coordination at the national level (governance, roles, and information flow):**

- The Emergency National Command Center (ENCC) established by the Government, with support from development partners, is responsible for Lesotho’s COVID-19 emergency response.
- The command center is multisectoral and headed by the MOF. It includes principal secretaries, directors general and directors of relevant ministries. It has put together an Integrated Plan that will first deal with the preparedness and containment of the pandemic, and then address economic and social impact. The committee meets as frequently as the urgency requires, but no less than twice a week, to advise and support activities in a systematic manner and take evidencebased decisions related to response and impact mitigation of the pandemic. The Emergency National Command Center has a national PIU for the COVID-19 emergency response^[[65]](#footnote-65)^.

**Coordination at the county/ district/ local regions (governance, roles, and information flow):**

**When did they start?**

- Response started before the first case^[[66]](#footnote-66)^.
- First case 13/05/2020^[[67]](#footnote-67)^.

**Any changes/ gaps and lessons learnt?**

- Past experience with disasters helps strengthen covid 19 response^[[68]](#footnote-68)^.

1. **LIBERIA**

**Coordination at the national level (governance, roles, and information flow):**

- The Liberian President George Weah declared a nationwide state of emergency on 8 April 2020, with mandatory lockdown in several regions, including the capital, Monrovia, to combat the spread of the coronavirus. The Liberian Senate has since approved an extension of the state of emergency throughout the country for 90 days in order to control the spread of the coronavirus. Among the immediate measures adopted, the President banned the entry into Liberia of persons from countries where there were more than 200 confirmed cases of COVID-19.
- The Government, in coordination with the United Nations (UN), Donor Partners, the Ministry of Health and the National Public Health Institute of Liberia (NPHIL) put in place the National Multi-sectoral Response Plan (NMRP) to COVID-19.
- The President also appointed Ms Mary Broh to act as National Coordinator of the Executive Committee on coronavirus (ECOC) response in Liberia. In this role, Ms. Broh is to oversee a unique set of national strategic objectives to defeat the coronavirus disease.
- The President again appointed Finda Bundoo as National Compliance Officer, responsible for overseeing the overall allocation and disbursement of logistics and resources for the COVID-19 response. In consultation with the National Response Coordinator, Ms. Bundoo is to ensure the timely delivery of supplies for the control of COVID-19 in the country and account for them.
- 200 contact tracers have been recruited, trained and deployed on an emergency basis with the assistance of the United Nations. They are responsible for identifying those who have been in contact with infected persons and, in case they have symptoms, testing them. The contact tracers are also responsible for educating the community about how to prevent infections^[[69]](#footnote-69)^.

**Coordination at the county/ district/ local regions (governance, roles, and information flow):**

**When did they start?**

- 16/03/2020^[[70]](#footnote-70)^.

**Any changes/ gaps and lessons learnt?**

- Ebola contact tracing lessons inform COVID-19 response^[[71]](#footnote-71)^.

1. **MADAGASCAR**

**Coordination at the national level (governance, roles, and information flow):**

- COVID-19 pandemic coordination is still ensured by three existing coordination bodies (political through the Presidency, strategic under the lead of with the Prime Minister and operational with the COVID-19 Operational Command Center (CCOC/MoH)). In support to these bodies, a military platform was created aiming to support mainly the digitalization of cases and hospital bed attribution^[[72]](#footnote-72)^.

**Coordination at the county/ district/ local regions (governance, roles, and information flow):**

**When did they start?**

- **20/03/2020^[[73]](#footnote-73)^**

**Any changes/ gaps and lessons learnt?**

1. **MALAWI**

**Coordination at the national level (governance, roles, and information flow):**

- The Special Cabinet Minister’s Committee on COVID-19 is the high-level coordination structure overseeing cross-Government preparedness and response activities of the COVID-19 outbreak.
- The National Disaster Preparedness and Relief Committee (NDPRC) chaired by the Chief Secretary to Government comprising of Permanent Secretaries from all government ministries will provide policy guidance and leadership in implementation of the plan.
- The Humanitarian response Committee composed of directors of government departments and heads of humanitarian partners, NGOs and CSOs will provide technical support and advice to the NDPRC in implementation of the plan.
- The Ministry of Disaster Management Affairs and Public Events and the UNRCO are responsible for facilitating resource mobilization, effective and efficient implementation of COVID-19 preparedness and response for UN- Agencies and development partners through the Humanitarian Country Team (HCT). The Ministry of Health is the technical lead institution for implementing COVID-19 preparedness and response activities and will provide all the necessary technical support and expertise
- At the health ministerial level, a multi-sectoral Health Cluster Committee reviews and endorses the decisions provided by the Health Emergency Technical Committee (HETC). Both Committees include bilateral and multilateral partners such as WHO, UNICEF, FAO, USAID, CDC, both at local and international level and meet weekly to coordinate preparedness and response. 4UNHCR should co-lead the Protection cluster; however, UNHCR Malawi indicated that it does not have the capacity at the local level to provide support to the cluster. In the event of a major emergency, UNCHR will assume its global responsibilities and provide leadership to the cluster in support of UNICEF.
- A taskforce on COVID-19 is responsible for developing the technical guidelines, interventions, preparedness plans and budget as well as ensuring operational readiness for any COVID-19 outbreak. The taskforce sits at the Public Health Institute of Malawi (PHIM) and feeds into the HETC and Health Cluster Committees.
- An Incident Management System has been set up at PHIM to ensure efficient coordination of activities with the following functions:
- Health Operations and Technical Expertise of Surveillance, Laboratory, PoE, IPC, WASH, Case Management, Risk Communication
- Partner Coordination of Resource mobilization, including technical and financial
- Logistics and Supplies of Health Procurement and Inventory, Operational Support
- Planning and Information o Surveillance and Early Warning, Monitoring and Evaluation
- Administration and Finance o Human Resource, Financial Management
- The Office of the President and Cabinet set up a Special Cabinet Minister’s Committee on COVID-19 on 7th March 2020 as high-level coordination structure overseeing cross Government preparedness and response activities of the COVID-19 outbreak.
- The committee comprises the following Ministries: Health (Chairperson); Disaster Management Affairs and Public Events; Minister of Finance and Economic Planning; Education Science and Technology; Homeland Security; Defence; Industry, Trade and Tourism; Agriculture, Irrigation and Water Development; Foreign Affairs and International Cooperation. The National Disaster Preparedness and Relief Committee (NDPRC) chaired by the Chief Secretary to Government comprising of Permanent Secretaries from relevant ministries will provide policy guidance and leadership in implementation of the plan.
- The National Disaster Preparedness and Relief Technical Committee will provide technical support and advice to the NDPRC in implementation of the plan. The office of the UN Resident Coordinator (UNRCO) is responsible for facilitating resource mobilization, effective and efficient implementation of COVID-19 preparedness and response for UN- Agencies and development partners through the Humanitarian Country Team (HCT)^[[74]](#footnote-74)^.

**Coordination at the county/ district/ local regions (governance, roles, and information flow):**

- At the district level, similar structures of from the health cluster down are replicated^[[75]](#footnote-75)^.


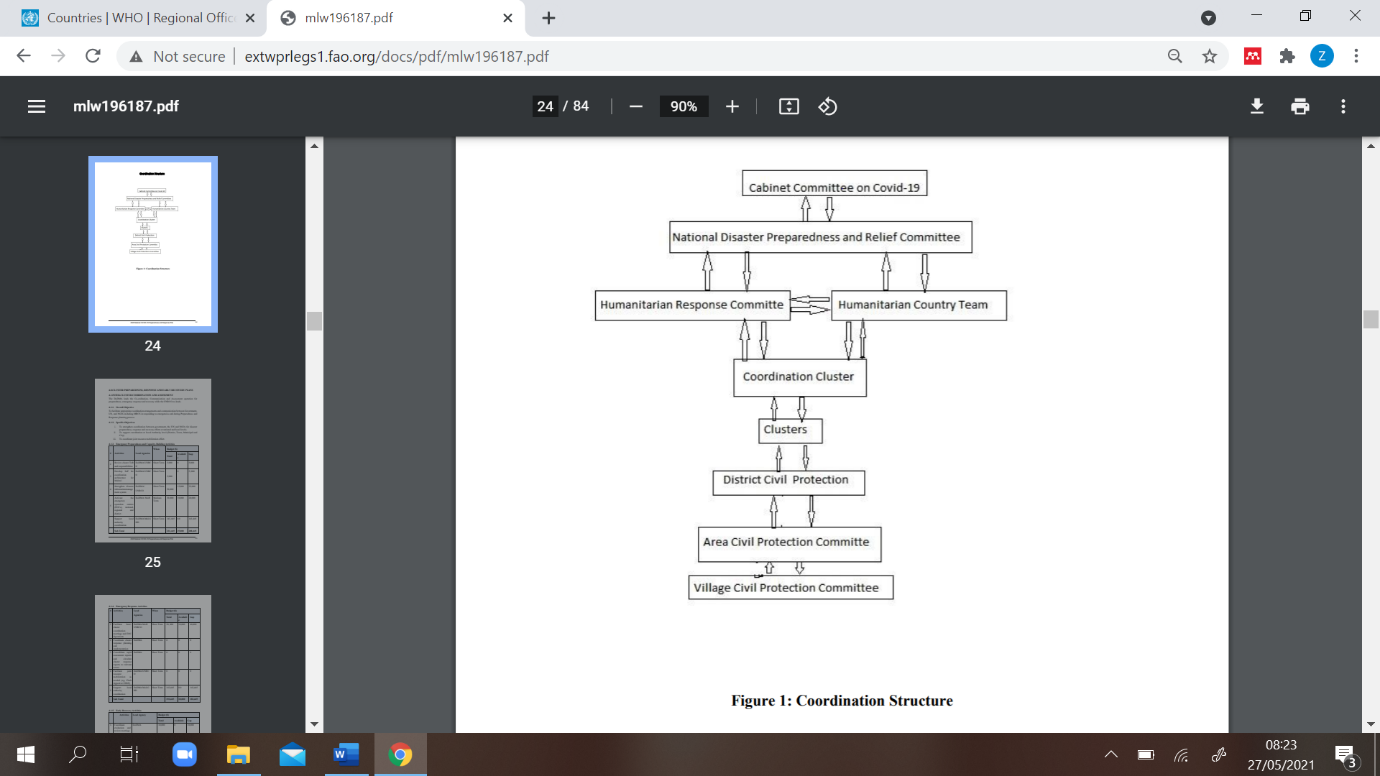


**When did they start?**

- **2/4/2020^[[76]](#footnote-76)^.**

**Any changes/ gaps and lessons learnt?**

1. **MALI**

**Coordination at the national level (governance, roles, and information flow):**

- Even before the appearance of confirmed cases of COVID-19 in Mali, the government constituted a Defence Council presided over by the President to respond to the threat of the pandemic. On March 11, 2020, the country was put on high alert through the announcement of a number of measures of restriction listed below. Once the first two cases were recorded, the measures were reinforced with a curfew from 9pm to 5am on March 26, 2020, and the closure of all educational institutions. Measures were also announced to mitigate the impact of the restrictions on the economy and livelihoods^[[77]](#footnote-77)^.
- In February 2020, the Malian Ministry of Public Health developed an Action Plan on the prevention and control of the disease that focused towards four main areas: *i) Prevention, ii) Communication, iii) Capacity building and iv) Prevention and case management*^[[78]](#footnote-78)^.
- Mali created the COVID-19 TCC under the leadership of the prime Minister’s office and the SAC to the MoH to provide strategic orientation and evidence-based guidelines for the management of the COVID-19 pandemic.
- The TCC oversees and coordinates general multisectoral response activities for the country (e.g., police, border security, veterinary services, national communications, academia and civil society). It is co-directed by the Coordinator of National COVID-19 Response and the Coordinator of the National Platform for Disaster Risk Management led by the Ministry of Homeland Security and Civilian Protection. TCC recommendations are reviewed and adopted during inter-ministerial meetings led by the Prime Minister. The SAC, chaired by UCRC leadership, is composed of scientists and clinical researchers who meet weekly to assess the status of the epidemic and make recommendations to the MoH on COVID-19 operational issues, including local standards of care and research priorities. SAC representatives participate at the Higher Council of National Defense to advise the President of Mali on strategic decisions regarding COVID-19, including curfew, school closures and lockdowns. Within the MoH, a complementary committee of subject matter experts from different MoH departments was created to oversee control and surveillance.
- To finance rapid implementation of control measures, the government has created a National Fund to fight COVID-19. This fund is supported by the Malian government in conjunction with private sector donations. Its current balance is over US $8,563,300 and intended to cover the purchase of drugs, healthcare workers, personal protective equipment, hospital supplies and containment centres. In addition, CRNST was able to secure 229,000€ from The National Competitive Fund for Research and Technological Innovation, which is generated through tax revenue of 0.20% of collections. CNRST then set up a request for COVID-19 proposals, which received 41 applications within 2 weeks. Proposals included clinical studies on diagnosis, treatment and mental health; social and behavioural studies on socio-economic impacts and knowledge, attitudes and practices; evaluation of a new physiotherapeutic intervention; and innovations related to e-health surveillance.

**Coordination at the county/ district/ local regions (governance, roles, and information flow):**

**When did they start?**

- Response began before 1^st^ case^[[79]](#footnote-79)^.
- First case 25/03/2020^[[80]](#footnote-80)^.

**Any changes/ gaps and lessons learnt?**

- The public health response has been fairly structured, as exemplified by the rapid establishment of three treatment centres in Bamako. However, the research response has been less organised. In the setting of overtaxed capacity, social distancing and desire of multiple partners to rapidly implement studies addressing diverse issues, the limited research infrastructure is challenged to effectively engage and coordinate with its enthusiastic partners and separate research groups with interests in Mali. Proposed COVID-19 projects with UCRC address general epidemiology, potential natural and pharmaceutical interventions, prevention strategies, and the impact on healthcare personnel. Some studies are specific to Mali, while others are conducted through international networks. There is also a general emerging infection study that was being finalised and implemented when COVID-19 emerged. This study serves to collect observational data on COVID-19 patients while more specific protocols are implemented^[[81]](#footnote-81)^.

1. **MAURITANIA**

**Coordination at the national level (governance, roles, and information flow):**

- The Government has put in place an inter-ministerial Committee on 11 March presided by the Prime Minister to manage the response to the pandemic. The committee has appointed a number of sub-committees, all of which are responsible for dealing with specific aspects of the pandemic. The committee is tasked with the monitoring of the spread of the virus nationally and internationally and coordinating the appropriate response. It opened a hospital with 100 beds on 31 March, used for the isolation of suspected COVID-19 patients.The Committee assigned a multidisciplinary medical team to monitor persons placed in sanitary confinement.
- On 5 April, the Justice Interior and Defense Committee of the National Assembly *(La commission de la justice, de l'intérieur et de la défense à l'Assemblée Nationale)* discussed the adoption of an Act which would authorise the government, pursuant to Article 60 of the Constitution, to take all necessary measures to respond to the pandemic. The President would hence have the power to take all necessary and appropriate measures by ordinance, during a defined period of time to manage the response to CVOID-19^[[82]](#footnote-82)^.

**Coordination at the county/ district/ local regions (governance, roles, and information flow):**

**When did they start?**

- 13/03/2020^[[83]](#footnote-83)^

**Any changes/ gaps and lessons learnt?**

- Lack of early coordination of state and non-state actors^[[84]](#footnote-84)^.

1. **MAURITIUS**

**Coordination at the national level (governance, roles, and information flow):**

- The COVID-19 response in Mauritius benefitted from strong leadership and highest political engagement. A High-Level COVID-19 Committee, chaired by the Prime Minister, was instituted on 31 January 2020 to monitor the local and international epidemiological situations and to rapidly share key information among the different ministries. The committee was composed of ministers in charge of Health and Wellness; Foreign Affairs, Regional Integration and International Trade; Finance, Economics, Planning and Development; Tourism; as well as the WHO Representative, the Secretary to Cabinet and Head of Civil Service, medical technical advisors and other key stakeholders.
- This whole-of government approach enabled a timely and informed decision-making for a coordinated and scaled-up national response. In addition to this, an intersectoral committee was established at the level of Ministry of Health and Wellness (MoHW), which monitored the evolution of the epidemiological situation as well as the daily activities related to the operational plan. It also elaborated guidelines and standard operating procedures when needed.
- Numerous protocols were devised for the different processes of case management, including transfer to intensive care unit, oxygenation, ventilation, discharge of recovered patients and taking charge of patients’ family members. Some of the protocols were the WHO protocols, whereas others were adapted to the local context, taking into consideration the capacities of the Mauritian healthcare system. Many of them were modified several times to adapt to the changes in resources and epidemiological context and integrate the lessons learnt^[[85]](#footnote-85)^.

**Coordination at the county/ district/ local regions (governance, roles, and information flow):**

**When did they start?**

- 18/03/2020^[[86]](#footnote-86)^

**Any changes/ gaps and lessons learnt?**

- The COVID-19 response in Mauritius, which is viewed as a success story, benefited from strong leadership, highest political engagement, and strong involvement of the WHO, the private sector and other key stakeholders.
- Another key success factor lies in clear, transparent and consistent communication, including feedback mechanisms to understand public perception, the result of which is good adherence by the population.
- Public health measures (quarantine, contact tracing, case investigation and isolation of contacts, mass testing) have also contributed to the success of the country in the fight against COVID-19.
- The Mauritian success story can be inspiring for other countries, especially regarding the importance of adapting measures to evolving knowledge and developing a clear and consistent communication policy so as to buy the adherence of the population.
- However, contextual factors (an island has a limited number of entry points) have also facilitated the implementation and success of these measures. Not all countries can expect similar results by copy pasting the Mauritian response strategy and should probably adopt a comprehensive policy acting on various factors to fight the pandemic^[[87]](#footnote-87)^.

1. **MOZAMBIQUE**

**Coordination at the national level (governance, roles, and information flow):**

- The Presidential Decree nº11/20205, of 30th March, declared a State of Emergency across the whole country, with the aim of implementing measures for the prevention and mitigation of Covid-19’s social impacts. The Decree nº12/20206, of 2nd April, article 36, approved administrative enforcement measures for the prevention and containment of the spread of the Covid-19 pandemic^[[88]](#footnote-88)^.

**Coordination at the county/ district/ local regions (governance, roles, and information flow):**

**When did they start?**

- **22/03/2020**

**Any changes/ gaps and lessons learnt?**

1. **NAMIBIA**

**Coordination at the national level (governance, roles, and information flow):**

- When COVID-19 emerged, the government showed strong leadership and ownership of the situation by activating a National Health Emergency Coordination Committee under the Ministry of Health and Social Services (MOHSS). The Namibian government has furthermore introduced the Incident Management System (IMS) and strengthened the functionality of the National Public Health Emergency Operation Centre (NPHEOC), which has become the central base from where all COVID-19 responders at the national level are operating from.
- The government brought all government sectors, development partners, private sector and civil society on board and developed a Multi-sectoral National Response Plan for COVID-19 in Namibia, which resulted into a whole-of-government, whole-of-society response campaign led by the Head of State, His Excellency Dr. Hage Geingob, with senior leadership of numerous government ministries. This response campaign is comprised of several technical pillars, including the *Country Co-ordination and Planning, Risk Communication and Community Engagement; Surveillance, Case investigation and rapid response; Points of Entry; National laboratory, Infection Prevention and Control; Case Management, Operations, Logistics and procurement; and Mental Health and Psychosocial support, Security and provision of essential services.*
- Efforts have been put in place to make sure all interventions are well coordinated, communicated for effective implementation^[[89]](#footnote-89)^.

**Coordination at the county/ district/ local regions (governance, roles, and information flow):**

**When did they start?**

- **First case-13/03/2020^[[90]](#footnote-90)^.**

**Any changes/ gaps and lessons learnt?**

1. **NIGER**

**Coordination at the national level (governance, roles, and information flow):**

- The Government of Niger has developed a national COVID-19 Emergency Preparedness and Response Plan for a total budget of 102,839,078,927 FCFA, comprised of the following 5 strategic axes: reinforcement of coordination; strengthening of epidemiological surveillance, strengthening of health services capacities; reinforcement of risk communication and community engagement; creation of isolation sites. Eight committees have been created to implement the plan:
- Coordination, planning and monitoring (including official measures for restrictions on movements and activities)
- Risk Communication and Community Engagement (limit non-essential movements and activities, practice social distancing, and enhance hygiene practices)
- Epidemiological surveillance (line-listing, contact-tracing, investigation, daily reporting and trend analysis)
- Laboratory and research (swab testing and case confirmation)
- Infection Prevention and Control, Hygiene and Sanitation (SOPs, designated triage and treatment sites, Personal Protective Equipment)
- Case management (including artificial respiration support for most severe cases)
- Response et monitoring (including psycho-social care)
- Logistics (procurement, local production, storage, distribution of supplies and equipment as per itemized and quantified list)^[[91]](#footnote-91)^.

**Coordination at the county/ district/ local regions (governance, roles, and information flow):**

**When did they start?**

- **19/03/2020**^[[92]](#footnote-92)^.

**Any changes/ gaps and lessons learnt?**

- Strong commitment of the government and technical and financial partners to support the fight against the pandemic. Also, the involvement of different sectors, experts and layers of civil society has helped to improve decision-making and community engagement.
- Increased communication has made it possible to face several challenges related to the management of the pandemic.
- These lessons learned need to be capitalized and strengthened to ensure the success of the vaccination campaign Deploying multidisciplinary expert teams to affected regions due the influx of people through land borders proved crucial in the response.

1. **NIGERIA**

**Coordination at the national level (governance, roles, and information flow):**

- Following reports of the coronavirus disease in Wuhan, China in December 2019, the NCDC published a notification of a new virus on its website on January 7, 2020. Subsequently, on January 26, 2020, the NCDC established a multisectoral National Coronavirus Preparedness Group (NCPG) in order to ensure a cohesive and effective coordination of the country’s preparedness efforts. The NCPG met daily to review global COVID-19 epidemiology, assess the risk of spread, and initiate measures to strengthen the country’s preparedness for early detection and timely response in the event of a COVID-19 outbreak in Nigeria.
- An inter-ministerial Multisectoral Technical Working Group was inaugurated at the Federal Ministry of Health on January 31, 2020, to further strengthen preparedness. Measures instituted by the NCPG included strengthening in-country diagnostic capacity for the testing of COVID-19 by leveraging and optimising three existing laboratories within the NCDC molecular laboratory network and assessing existing infectious disease treatment centres with a focus on identifying gaps and developing plans for case management.
- Interim protocols and guidelines for case management of COVID-19 were developed while the Nigeria Pandemic Influenza Preparedness and Response plan was reviewed for relevance to COVID-19 response. Infection prevention and control (IPC) and case management trainings were conducted for frontline health care workers in designated treatment centres^[[93]](#footnote-93)^.

**Coordination at the county/ district/ local regions (governance, roles, and information flow):**

**When did they start?**

- **Response began before 1^st^ case^[[94]](#footnote-94)^**
- **First case-27/02/2020**^[[95]](#footnote-95)^

**Any changes/ gaps and lessons learnt?**

- Lessons from Ebola epidemic informed COVID-19 response^[[96]](#footnote-96)^.

1. **RWANDA**

**Coordination at the national level (governance, roles, and information flow):**

- As part of its COVID response, Rwanda announced the formation of a Joint Task Force on 3 March 2020, which created the Rwandan Coronavirus National Preparedness and Response Plan, with the primary objective of “stopping the human-to-human transmission of the virus and caring for those affected
- The government of Rwanda has activated the National Epidemic Preparedness and Response Coordination Committee, which appointed the COVID-19 National Steering Committee to oversee the coordination of Joint Task Force activities, grouped into epidemiology operations, administrative and logistics communication, and planning units. The Rwanda Biomedical Centre (RBC) and the MOH are the nation’s central health implementation agencies who have collaborated to develop a six-month National COVID-19 Preparedness and Response Plan. This plan includes four phases of country response: Pre-epidemic; sporadic cases dealing with limited numbers of imported cases from affected countries; single clusters of cases responding to local transmission in a district, sector, or village; and community transmission involving more than one cluster.
- During the preparation phase, incident management concentrated on establishing a rapid response team (RRT) in districts and health units, with 480 trained staff, in addition to the creation of COVID-19 Standard Operating Procedures (SOPs).The SOPs guide the national response to COVID-19, including leadership structure, infection prevention, epidemiological surveillance, handling of clinical specimens, psychosocial support, case management, community mobilization, and managing misinformation. Importantly, the plan includes the critical step of forming a coordinated national COVID-19 Incident Management System^[[97]](#footnote-97)^.


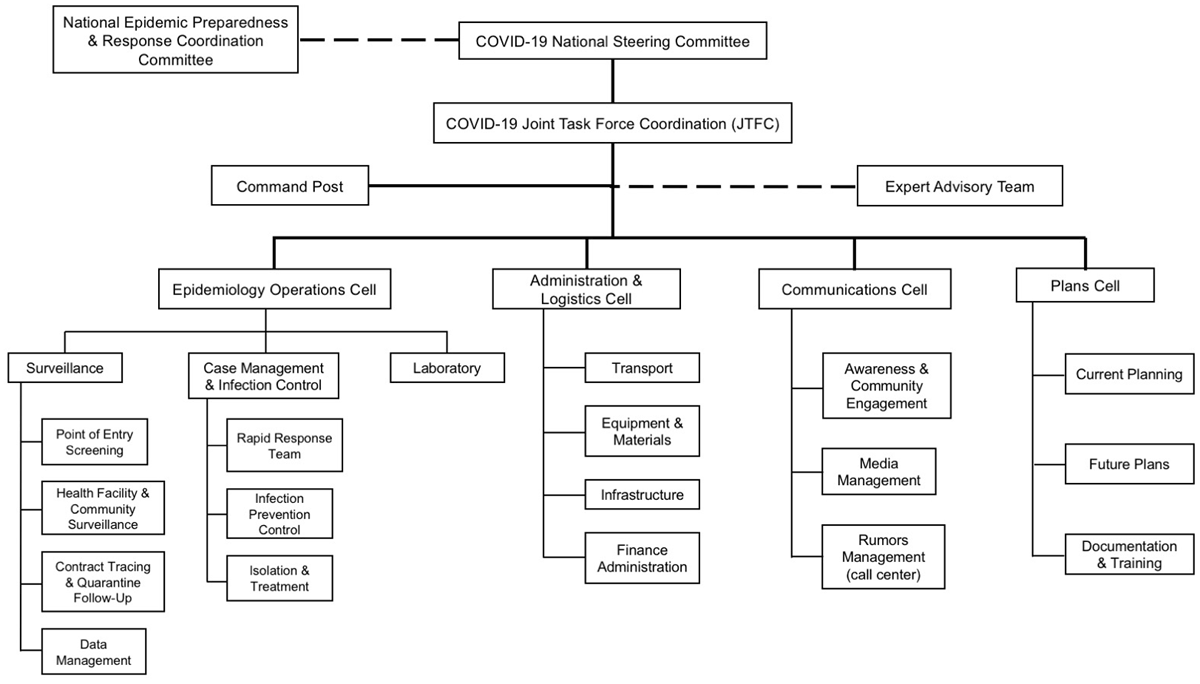


**Coordination at the county/ district/ local regions (governance, roles, and information flow):**

- The COVID-19 response in each of Rwanda’s 30 districts is led by the mayors, who coordinate the interventions for every aspect of infection control.

**When did they start?**

- **14/03/2020**^[[98]](#footnote-98)^

**Any changes/ gaps and lessons learnt?**

- Rwanda’s government and healthcare system has responded to the COVID-19 pandemic with innovative interventions to prevent and contain the virus. Importantly, the response has utilized adaptive and innovative technology and robust risk communication and community engagement to deliver an effective response to the COVID-19 pandemic.
- Much of Rwanda’s pandemic response has adopted or leveraged existing infrastructure from Ebola preparedness efforts in 2018–19, highlighting the advantages of comprehensive pandemic preparation experience for a country. For example, strategies for developing a National Preparedness Plan, training health workers and equipping health facilities, establishing dedicated treatment centers, conducting simulation exercises, educating the public, and screening extensively at national points of entry during the Ebola efforts have served as a strong foundation for the COVID-19 response^[[99]](#footnote-99)^.

1. **SAO TOME AND PRINCIPE**

**Coordination at the national level (governance, roles, and information flow):**

- In April 2020, the Government turned to the United Nations Development Programme (UNDP) for support in strengthening its national COVID-19 response capacity. UNDP, in close collaboration with the Ministry of Health, designed a comprehensive capacity development project that included a wide spectrum of young professionals to help strengthen key areas in the health sector of São Tomé and Príncipe, identified as critical in mitigating the impacts of the pandemic^[[100]](#footnote-100)^

**Coordination at the county/ district/ local regions (governance, roles, and information flow):**

**When did they start?**

- **First Case-6/4/2020^[[101]](#footnote-101)^**

**Any changes/ gaps and lessons learnt?**

1. **SENEGAL**

**Coordination at the national level (governance, roles, and information flow):**

- The country’s Health Emergency Operations Center, which is now coordinating the COVID-19 response at an operational level, was set up after the 2014 Ebola outbreak. When COVID-19 first emerged as a concern in January, the center began by assessing the response capacities of the country, according to Imboua, and then used the results of its assessment to build the capacity of actors at all levels^[[102]](#footnote-102)^.

**Coordination at the county/ district/ local regions (governance, roles, and information flow):**

**When did they start?**

- **2/3/2020^[[103]](#footnote-103)^**

**Any changes/ gaps and lessons learnt?**

- Health officials working in the country attribute its success to experience, preparation, and a timely government response.
- Senegal, like all other countries in sub-Saharan Africa, is used to managing outbreaks and has the experience and capacity to respond. The experience gained from the Ebola outbreak has been useful in triggering preparedness and response interventions.

1. **SEYCHELLES**

**Coordination at the national level (governance, roles, and information flow):**

- Seychelles has attained universal health coverage (UHC) with the State providing free and universal access to health care for all Seychellois. However, COVID-19 pandemic threatens to reverse these gains and risks the sustainability of Seychelles universal health coverage. Cognizant of this threat, the Government is implementing measures under the COVID-19 National Response Plan, which have been effective in preventing community transmissions. The first case of COVID-19 in Seychelles was confirmed on 14th March and by the 6th of April, there were 11 confirmed imported cases. The goal of the COVID-19 response plan is to prevent, promptly detect and effectively respond to any COVID-19 outbreak to reduce morbidity and mortality in the country^[[104]](#footnote-104)^.

**Coordination at the county/ district/ local regions (governance, roles, and information flow):**

**When did they start?**

- **First case-14/3/2020**^[[105]](#footnote-105)^

**Any changes/ gaps and lessons learnt?**

1. **SIERRA LEONE**

**Coordination at the national level (governance, roles, and information flow):**

- During Sierra Leone’s prevention phase, efforts were made to reactivate the community sensitisation structures used during the Ebola outbreak. Officials from the Ministry of Health and Sanitation visited and informed several community leaders nationwide. While this effort was commendable, it was not followed through. At best, subsequent efforts were not inclusive, as other major stakeholders – such as opposition political parties, parliamentarians, and local councillors (especially those from opposition areas) – were not adequately engaged.
- Initial response and coordination efforts were led by the Ministry of Health and Sanitation.  This effort was highly commended as almost all stakeholders, including the leadership of political parties, were engaged. Perhaps most importantly, the leadership of the Ebola Response team was invited and participated. Learning from the Ebola experience, a dedicated structure (Emergency Operation Centre/EOC) was constituted to coordinate all stakeholders.
- The initially applauded inclusive approach, which included all political persuasions, has been diluted by conflicting accusations between Government and the main opposition political party. The arrest and detention of the head of the National Ebola Response programme on allegations of attempted treason has resulted in opposition party members no longer participating in the EOC.

**Coordination at the county/ district/ local regions (governance, roles, and information flow):**

**When did they start?**

- **Response began before 1^st^ case**^[[106]](#footnote-106)^
- **First case – 31/3/2020**^[[107]](#footnote-107)^.

**Any changes/ gaps and lessons learnt?**

- **Inclusive stakeholder coordination**: It goes without saying that a coordinated and inclusive approach will help boost social mobilisation efforts and help contain the pandemic. Therefore, it is important for the government to review its coordination strategy to include all stakeholders including opposition parties. The lessons from the Ebola crisis clearly suggest that there was significant acceptance of the social mobilisation messages when political parties and community leaders joined forces with the government.
- **An adaptive and long-term response**: Finally, while the swift attempt by government to prepare a response programme is highly commendable, given the evolving nature of the pandemic, backed by the additional data now available, it is important for the government to review QAERP to not only reflect the new realities, but the underlying challenges the country was facing even before the crisis^[[108]](#footnote-108)^.

1. **SOUTH AFRICA**

**Coordination at the national level (governance, roles, and information flow):**

- The South African Department of Health coordinates national response to COVID-19 pandemic, which in turn engages through Cabinet’s National Command Council for decision-making and coordinating the management of interventions by various institutions and individuals^[[109]](#footnote-109)^.
- A multisectoral approach to containing and mitigating the spread of SARS-CoV-2 was instituted, led by the South African National Department of Health. A National COVID-19 Command Council was established to take government-wide decisions. An adapted WHO COVID-19 strategy for containing and mitigating the spread of the virus was implemented by the National Department of Health. The strategy included the creation of national and provincial incident management teams (IMTs), which comprised of a variety of work streams, namely, governance and leadership; medical supplies; port and environmental health; epidemiology and response; facility readiness and case management; emergency medical services; information systems; risk communication and community engagement; occupational health and safety and human resources^[[110]](#footnote-110)^.

**Coordination at the county/ district/ local regions (governance, roles, and information flow):**

**When did they start?**

- **First case – 5/3/2020**^[[111]](#footnote-111)^

**Any changes/ gaps and lessons learnt?**

- The following were the most salient lessons learnt between March and September 2020: strengthened command and control were achieved through both centralised and decentralised IMTs; swift evidenced-based decision-making from the highest political levels for instituting lockdowns to buy time to prepare the health system; the stringent lockdown enabled the health sector to increase its healthcare capacity. Despite these successes, the stringent lockdown measures resulted in economic hardship particularly for the most vulnerable sections of the population^[[112]](#footnote-112)^.

1. **SOUTH SUDAN**

**Coordination at the national level (governance, roles, and information flow):**

- As part of the support, WHO constructed the Public Health Emergency Operation Centre, the first of its kind in South Sudan and the Dr John Garang’ multi-purpose Infectious Disease Unit; as well as strengthened the capacity of the laboratory to test infectious diseases such as measles, Ebola and COVID-19 among others.
- In addition, the Ministry of Health in collaboration with WHO and other partners launched a national wide preparedness and response plan for COVID-19 aimed to support *coordination and planning with other actors, surveillance, rapid response teams and case investigation, case management, laboratory testing, risk communication, community engagement and social mobilization, and infection prevention and control*^[[113]](#footnote-113)^.

**Coordination at the county/ district/ local regions (governance, roles, and information flow):**

**When did they start?**

- **First case – 5/4/2020**^[[114]](#footnote-114)^.

**Any changes/ gaps and lessons learnt?**

1. **TOGO**

**Coordination at the national level (governance, roles, and information flow):**

- The Government has adopted a number of measures to prevent and mitigate the spread of the pandemic.
- A National Coordination of the response to COVID-19 has been created and, in the health field, actions are taken to ensure the health care of people who are tested positive with COVID-19, to promote individual protection measures such as hand washing, the use of masks, the use of hydro alcoholic gel, and the respect of social distancing^[[115]](#footnote-115)^.

**Coordination at the county/ district/ local regions (governance, roles, and information flow):**

**When did they start?**

- **First case – 6/3/2020**^[[116]](#footnote-116)^.

**Any changes/ gaps and lessons learnt?**

- K

1. **UGANDA**

**Coordination at the national level (governance, roles, and information flow):**

- The government appointed an emergency response team to coordinate the response across six pillars. These are: *governance and leadership; surveillance and laboratory; case management; logistics; risk communication, social mobilization, and community engagement; and mental and psychosocial support*.
- While the Ministry of Health (MOH) was responsible for policy and strategy, it coordinated with the Office of the Prime Minister on the strategic and operational command of the response, as well as a series of cross-cutting functions, such as planning, budgeting, and partner coordination. The already existing district surveillance teams and District Task Forces (DTF) were immediately called into play to respond to the virus in their jurisdictions.
- A multisectoral approach financed, managed, and combatted the response. Funds for surveillance, sample collection, and contact tracing for districts were channelled through local governments, while those for enforcing lock-down measures and quarantine were channelled through Ministry of Internal Affairs, Department of Defence.
- Once the lockdown was initiated, the GoU quickly proceeded to set up the institutional arrangements needed to adequately respond to COVID-19. The Ugandan National Security Council set up a multisectoral National Task Force (NTF), with representatives from the Office of the Prime Minister, Health, Internal Affairs, Defence, Works and Transport, and Trade and Industry, as well as information and communications technology sectors, Kampala Capital City Authority (KCCA), and the private sector.


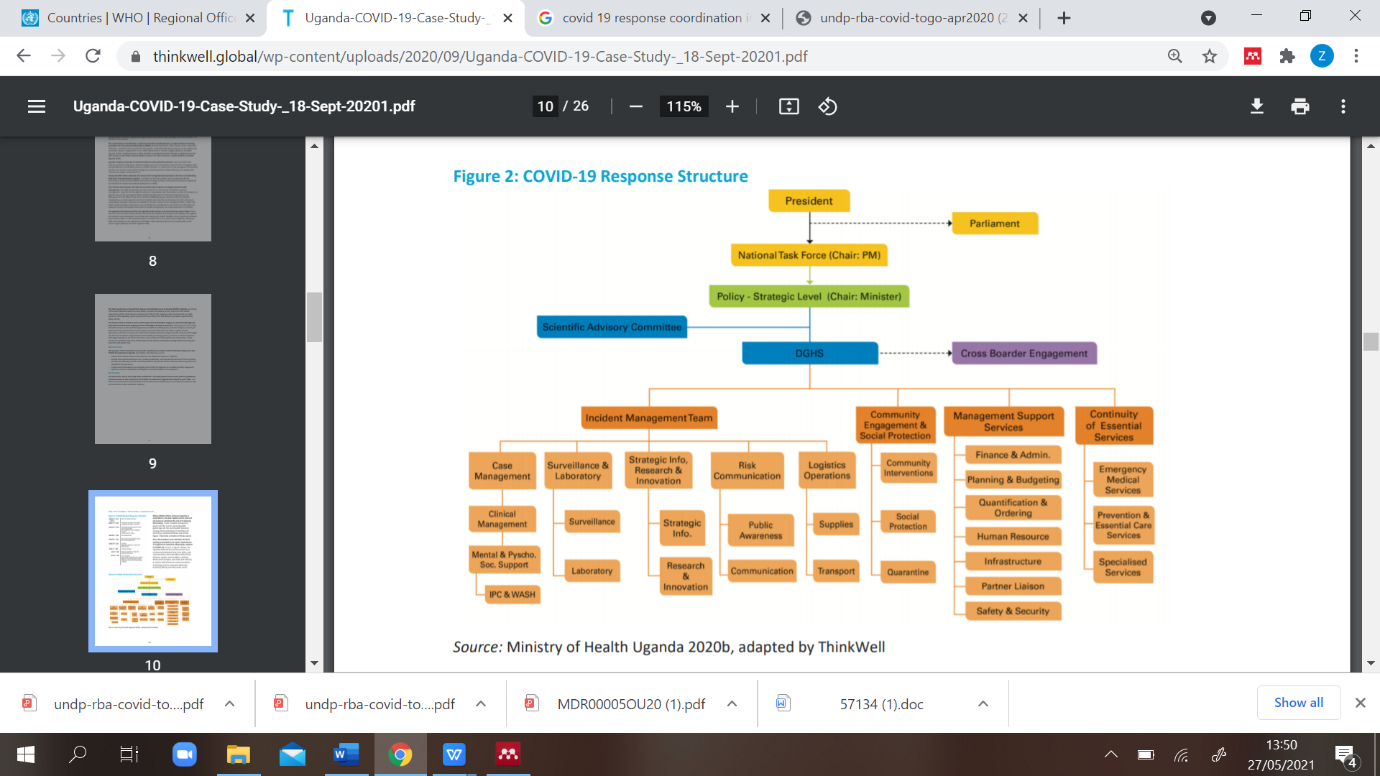


**Coordination at the county/ district/ local regions (governance, roles, and information flow):**

**When did they start?**

**Any changes/ gaps and lessons learnt?**

- The health sector was given a smaller proportion of total funding for the response than expected. As a result, important challenges impeded full implementation of COVID-19 preparedness and response activities^[[117]](#footnote-117)^.

1. **UNITED REPUBLIC OF TANZANIA**

**Coordination at the national level (governance, roles, and information flow):**

- The Tanzanian Government formed three committees tasked with leading the fight against the COVID-19 pandemic. The first committee was the National Task Force headed by the Prime Minister that comprised of the Minister of Health, Ministers from other relevant ministries, and the Chief Government spokesperson. The second committee was the Committee of Permanent Secretaries headed by the Chief Secretary and comprising of permanent secretaries from relevant ministries, whose played an advisory role in the National Task Force. The third committee was the Technical Committee which was tasked with provision of technical advice to the Committee of Permanent Secretaries.
- The Technical Committee was led by the Permanent Secretary, Ministry of Health, Community Development, Gender, the Elderly and Children who delegated this authority to the Chief Medical Officer. The Technical Committee had 11 sub-committees including operations, finance and administration, planning, logistics, case management, surveillance, testing, points of entry, health education, water, sanitation, and hygiene (WASH), and research. These sub-committees worked closely with the regional and district disaster management committees to maintain a well-coordinated response from the national to district level.
- The three committees were set up to facilitate a well-coordinated COVID-19 response that supported rapid decision making and implementation required to control the pandemic. These committees essentially formed a decision loop from district level to the National Task Force, which was the ultimate COVID-19 response decision making entity. The communication loop brought together the operational aspect of the response (Technical Committee) together with the policy level (National Task Force and Committee of Permanent Secretaries) to fast-track decision making, planning and implementation that would not be possible under normal circumstances^[[118]](#footnote-118)^.

**Coordination at the county/ district/ local regions (governance, roles, and information flow):**

**When did they start?**

- First case-16/3/2020^[[119]](#footnote-119)^.

**Any changes/ gaps and lessons learnt?**

- Denial of the seriousness of the disease^[[120]](#footnote-120)^.

1. **ZAMBIA**

**Coordination at the national level (governance, roles, and information flow):**

- Zambia had applied a multisectoral national epidemic disease surveillance and response system^[[121]](#footnote-121)^
- Zambia national Public Health Institute (ZNPHI) – the Incident Command System has been established: *a) Case management team in-charge of the COVID-19 centres b) Surveillance teams c) Field workers for contact tracing d) Call centre staff who answer calls for alerts and follows up individuals in self quarantine by phone e) Laboratory services: 4 laboratories running the samples f) Health promotion/risk communication g) Team to develop guidelines, SOP’s, training materials & conduct trainings*
- Designated MOH isolation centres until the COVID-19 test is negative: *a) 2 main ones in Lusaka b) Each Province has at least 1 isolation center c) Active surveillance d) All admitted patients medical and paeds e) All admitted patients with COVID-19 –obs/gynae & surgery f) All mortalities BIDs*^[[122]](#footnote-122)^.

**Coordination at the county/ district/ local regions (governance, roles, and information flow):**

**When did they start?**

- **Response began before first case**^[[123]](#footnote-123)^.
- **First case – 18/3/2020**

**Any changes/ gaps and lessons learnt?**

1. **ZIMBABWE**

**Coordination at the national level (governance, roles, and information flow):**

- On 19 March 2020, the president of Zimbabwe, Emmerson Dambudzo Mnangagwa through the Statutory Instrument 76 of 2020 on Civil Protection (Declaration of State of Disaster: Rural and Urban Areas of Zimbabwe) declared COVID-19 as a national disaster. The government established a National COVID-19 Response Taskforce headed by the second Vice President Kembo Mohadi. This taskforce consists of representatives from different ministries and is further divided into subcommittees that are tasked to monitor the pandemic situation and coordinate the response to the crisis. It was also tasked with mobilising financial resources locally and internationally to cushion the country from the negative impacts of the pandemic^[[124]](#footnote-124)^.

**Coordination at the county/ district/ local regions (governance, roles, and information flow):**

**When did they start?**

- First case-20/3/2020^[[125]](#footnote-125)^.

**Any changes/ gaps and lessons learnt?**

1. Since the outbreak of COVID-19 pandemic in Zimbabwe on 20 March 2020, Many Zimbabweans have suffered a double tragedy. The Zimbabwean government is led by people with military background who can be described as men of strategies.
2. *Transparency*: Although the government of Zimbabwe has not been one of the most transparent entities, extraordinary times call for extraordinary action and this pandemic is one of such instances. The government can make available information on the number of cases, where and when the tests were taken, resources allocated to combat the disease and how they have been utilised, challenges facing the government in its responses and changing measures being implemented to keep up with the changing trends and emerging information about the disease. The government should also make public procurement processes, donations being received, efforts to make vaccine available when it is approved as well as how the government intends to cushion the citizens from the short- and long-term impacts of the disease.
3. *Effective and efficient response*: while the government has been implementing other globally popular approaches such as lock-downs, encouraging citizens to sanitize and keeping social distancing, there are issues that are particularly unique to Zimbabwe that requires the government to craft policies and adopt measure that will offer more practical solutions to the people. For example, given the already struggling economic situation in the country, the government can reach out to other development partners to help it provide face masks which are critical to reducing the spread of the disease yet very elusive especially for low-income earners [[16](https://www.ncbi.nlm.nih.gov/pmc/articles/PMC7648907/#R16)]. The government can also reach out to the military production unit to support in the production of reusable facemasks that can be distributed to the citizens. In addition, makeshift quarantine locations, testing points as well as improve sanitation and water delivery services can be provided more in crowded urban areas and this can be done with the support of the national youth service and the military.
4. *Show leadership in addressing the growing grievances*: Amid the pandemic, the government is having to deal with growing opposition from political, professional and community organisations over different concerns. For example, the strikes called by health practitioners demanding better pay and improved working environment comes at a critical time when the government needs more personnel in dealing with the pandemic. In addressing the grievances by frontline workers, the government needs to show leadership and not its coercive powers given that some of the concerns raised by these health workers are genuine and have a bearing on whether the country can overcome the challenges of the disease. Similarly, the on -going mass action called by activists demanding accountability could have been subverted should the government responded proactively to allegations of corruption especially with COVID-19 resources.
5. *Medical cost*: there is no doubt that one of the main concerns among Zimbabweans is whether they are able to afford the medical cost for testing and treating of COVID-19. This requires serious government attention as the issue of cost for treatment is also associated with the question of accessibility and availability of sufficient medical practitioners to provide treatment to the people. To solve this problem, the government will be required to adopt a multi -dimensional approach. For example, a robust public-private partnership initiative or a memorandum of understanding where private entities can collaborate with the government or exercise social corporate responsibility and pool recourse to help subsidise the cost of treatment or testing for the citizens. Insurance companies, both public and private can include COVID-19 treatment in their most basic premium.
6. *Re-evaluate the existing strategies periodically*: While this pandemic has put many governments to test over their ability to effectively respond to public health crises, most governments seem to be trapped with few response strategies which clearly appear to be ineffective in most countries. From measuring temperatures, to claims of contact tracing, and from alleged mass testing to availability of facilities, these strategies may have worked elsewhere but their implementation in developing countries does not appear to be working. For example, contact tracing requires the use of sophisticated technology, adequate personnel, appropriate intelligence to effectively implement. Similarly, mass testing requires sufficient kits, personnel, and standby facilities where those infected can be quarantined. So far, the Zimbabwean government which claims to be using the above strategies has not proved that it has sufficient capacity to adopt these measures. Therefore, the government can re-evaluate some of these approaches and replace them with cheaper, effective, and easily adaptable approaches.
7. *Bring others on board*: Zimbabwe’s governance structure is one that can be considered to be highly centralised. This kind of governance structure has increasingly become less popular especially in the wake of emerging public administration reform measures that brought about principles such as New Public Management and Good Governance. The two approaches emphasize decentralisation not just of functions but also decision making. The current pandemic offers a unique opportunity to adopt similar measures that will see increased involvement of different actors at the national, regional and the local levels. Apart from a recommended increase in inter-governmental relations, the central and local authorities can work closely irrespective of their political affiliations to curb the spread of the disease. Local response units can be established to work closely with National COVID-19 Response Taskforce based in the capital city of Harare. These local units are able to communicate valuable information about the realities on the ground ranging from preparedness in terms of health facilities, compliance to government guidelines and other emerging issues that can hinder the effectiveness of government response. The local units will act as a source of valuable information that can be used in the formulation, readjustment and monitoring of policies and government efforts to combat the disease^[[126]](#footnote-126)^.

1. “(No Title),” accessed May 27, 2021, https://www.icao.int/Security/COVID-19/StateActions/Algeria_EN.pdf. [↑](#footnote-ref-1)
2. “Spotlight Algeria ’ s Response to COVID-19 : An Ongoing Journey,” 2021, 2020. [↑](#footnote-ref-2)
3. “Coronavirus | Angola-State of Public Calamity and State of Emergency - Lexology,” accessed May 25, 2021, https://www.lexology.com/library/detail.aspx?g=63b71d23-b66f-4690-adbd-567d1ee8bffc. [↑](#footnote-ref-3)
4. “(No Title),” accessed May 25, 2021, https://www.unicef.org/media/74516/file/Angola-COVID19-Sitrep-24-April-2020.pdf. [↑](#footnote-ref-4)
5. “UNDP Angola,” 2020, 10027. [↑](#footnote-ref-5)
6. “Angolan Government Relying on Partner Assistance to Fight Covid-19 | Center for Strategic and International Studies,” accessed May 25, 2021, https://www.csis.org/analysis/angolan-government-relying-partner-assistance-fight-covid-19. [↑](#footnote-ref-6)
7. “Angola: Confronting the COVID-19 Pandemic and the Oil Price Shock,” accessed May 25, 2021, https://www.imf.org/en/News/Articles/2020/09/18/na-angola-confronting-the-covid-19-pandemic-and-the-oil-price-shock. [↑](#footnote-ref-7)
8. “Angolan Government Relying on Partner Assistance to Fight Covid-19 | Center for Strategic and International Studies.” [↑](#footnote-ref-8)
9. “Angolan Government Relying on Partner Assistance to Fight Covid-19 | Center for Strategic and International Studies.” [↑](#footnote-ref-9)
10. “Angolan Government Relying on Partner Assistance to Fight Covid-19 | Center for Strategic and International Studies.” [↑](#footnote-ref-10)
11. “UNICEF Angola COVID-19 Situation Report No.5, August 2020 - Angola | ReliefWeb,” accessed May 25, 2021, https://reliefweb.int/report/angola/unicef-angola-covid-19-situation-report-no5-august-2020. [↑](#footnote-ref-11)
12. Issideen Ayinla Osseni, “Benin Responds to Covid-19: Sanitary Cordon without Generalized Containment or Lockdown?,” accessed May 25, 2021, https://doi.org/10.1186/s41182-020-00235-6. [↑](#footnote-ref-12)
13. “Benin: Coronavirus (COVID-19) Situation Report No. 10 - Benin | ReliefWeb,” accessed May 25, 2021, https://reliefweb.int/report/benin/benin-coronavirus-covid-19-situation-report-no-10. [↑](#footnote-ref-13)
14. “Benin: Coronavirus (COVID-19) Situation Report No. 10 - Benin | ReliefWeb.” [↑](#footnote-ref-14)
15. Issideen Ayinla Osseni, “Benin Responds to Covid-19: Sanitary Cordon without Generalized Containment or Lockdown?,” *Tropical Medicine and Health* (BioMed Central Ltd., June 15, 2020), https://doi.org/10.1186/s41182-020-00235-6. [↑](#footnote-ref-15)
16. “UNDP Botswana,” n.d., 10027. [↑](#footnote-ref-16)
17. “Botswana’s Communities Stand Strong Against COVID-19 with USAID’s Assistance | News | Southern Africa Regional | U.S. Agency for International Development,” accessed May 25, 2021, https://www.usaid.gov/southern-africa-regional/fact-sheets/botswanas-communities-stand-strong-against-covid-19-usaids-assistance. [↑](#footnote-ref-17)
18. “(No Title),” accessed May 25, 2021, https://www.unicef.org/esa/media/6436/file/UNICEF Bostwana COVID-19 Tracker  19 June 2020 Final.pdf. [↑](#footnote-ref-18)
19. “Botswana Records First 3 Cases of Coronavirus - Health Minister | Reuters,” accessed May 25, 2021, https://www.reuters.com/article/health-coronavirus-botswana-idUSL8N2BK5AO. [↑](#footnote-ref-19)
20. T H E Burkina, Faso Sp, and P H C Team August, “COVID-19 Summary Update on Burkina Faso,” no. August (2020). https://thinkwell.global/wp-content/uploads/2020/05/COVID-19-Burkina-Faso-Update-December-2020-Final.pdf [↑](#footnote-ref-20)
21. Burkina, Sp, and August. [↑](#footnote-ref-21)
22. “MSF Supports Coronavirus COVID-19 Response in Cameroon,” accessed May 25, 2021, https://www.msf.org/msf-supports-covid-19-response-cameroon. [↑](#footnote-ref-22)
23. “MSF Supports Coronavirus COVID-19 Response in Cameroon.” [↑](#footnote-ref-23)
24. “Central African Republic - The Central African Republic Faces Health and Humanitarian Consequences of COVID-19 | Digital Situation Reports,” accessed May 25, 2021, https://reports.unocha.org/en/country/car/card/7jsVXwSZcV/. [↑](#footnote-ref-24)
25. “Central African Republic: Situation Report, 25 March 2021 - Central African Republic | ReliefWeb,” accessed May 25, 2021, https://reliefweb.int/report/central-african-republic/central-african-republic-situation-report-25-march-2021. [↑](#footnote-ref-25)
26. “Management of the COVID-19 Crisis in Chad | African Regional Organisation of the International Trade Union Confederation,” accessed May 25, 2021, http://www.ituc-africa.org/Management-of-the-COVID-19-Crisis-in-Chad.html. [↑](#footnote-ref-26)
27. “Chad Situation Report, 8 Jun 2020 - Chad | ReliefWeb,” accessed May 25, 2021, https://reliefweb.int/report/chad/chad-situation-report-8-jun-2020. [↑](#footnote-ref-27)
28. “Chad Situation Report, 8 Jun 2020 - Chad | ReliefWeb.” [↑](#footnote-ref-28)
29. “Chad Situation Report, 8 Jun 2020 - Chad | ReliefWeb.” [↑](#footnote-ref-29)
30. “Undp Comoros,” n.d., 567. [↑](#footnote-ref-30)
31. “THE COMOROS: Facing the Covid-19 Crisis | African Regional Organisation of the International Trade Union Confederation,” accessed May 25, 2021, http://www.ituc-africa.org/THE-COMOROS-facing-the-Covid-19-crisis.html. [↑](#footnote-ref-31)
32. “Comoros Confirms 1st Coronavirus Case,” accessed May 25, 2021, https://www.aa.com.tr/en/africa/comoros-confirms-1st-coronavirus-case/1825775. [↑](#footnote-ref-32)
33. “Mobilizing the COVID-19 Response in the DRC | PATH,” accessed May 25, 2021, https://www.path.org/articles/mobilizing-covid-19-response-drc/. [↑](#footnote-ref-33)
34. : “: COUNTRY REPORT / DRC 4 D E C E M B E R 2 0 2 0 V E R O N I Q U E D E G E O F F R O Y L E A N D R E M W E Z E DEC CVA REAL-TIME RESPONSE REVIEW: DEMOCRATIC REPUBLIC OF CONGO COUNTRY REPORT 2 DEC Coronavirus 2020 Appeal-Real Time Response Review-Country Report DRC,” n.d. [↑](#footnote-ref-34)
35. “Interview with Professor Muyembe, the Ebola and COVID-19 Response Coordinator in the DRC: ‘Community Engagement and Awareness-Raising Campaigns Are Key to Winning the Battle,’” accessed May 25, 2021, https://www.worldbank.org/en/news/feature/2020/05/19/interview-with-professor-muyembe-the-ebola-and-covid-19-response-coordinator-in-the-drc-community-engagement-and-awareness-raising-campaigns-are-key-to-winning-the-battle. [↑](#footnote-ref-35)
36. “UNDP Cote D ’ Ivoire,” 2020, 10027. [↑](#footnote-ref-36)
37. “The World Bank Cote d’Ivoire COVID-19 Strategic Preparedness and Response Project (SPRP) (P173813),” 2020, https://www.financialafrik.com/2020/03/31/covid-19-en-cote-divoire-le-premier-ministre-projette-une-. [↑](#footnote-ref-37)
38. “3 Questions with COVID-19 Coordinator Dr. Monique N’Guessan: Charting a Course for Côte d’Ivoire - Chemonics International,” accessed May 25, 2021, https://chemonics.com/blog/three-questions-with-covid-19-coordinator-dr-monique-nguessan-charting-a-course-for-cote-divoire/. [↑](#footnote-ref-38)
39. “UNDP Equatorial Guinea,” n.d., 10027. [↑](#footnote-ref-39)
40. Matthew De Bari and Scott Levi, “Oordination between State and Non - State Actors,” 2020. [↑](#footnote-ref-40)
41. “Equatorial Guinea: COVID-19 Situation Report – #10 (23 July- 26 August 2020) - Equatorial Guinea | ReliefWeb,” accessed May 25, 2021, https://reliefweb.int/report/equatorial-guinea/equatorial-guinea-covid-19-situation-report-10-23-july-26-august-2020. [↑](#footnote-ref-41)
42. Fikresus (Fikrejesus) Amahazion, “Examining Factors Contributing to Eritrea’s Successful COVID-19 Response,” *Open Journal of Social Sciences* 09, no. 02 (2021): 371–85, https://doi.org/10.4236/jss.2021.92025. [↑](#footnote-ref-42)
43. “ERITREA: Citizen Participation in Response to COVID-19 and Time for Renewal | African Regional Organisation of the International Trade Union Confederation,” accessed May 25, 2021, http://www.ituc-africa.org/ERITREA-Citizen-participation-in-response-to-COVID-19-and-time-for-renewal.html. [↑](#footnote-ref-43)
44. “NATIONAL EMERGENCY MANAGEMENT COORDINATION STRUCTURES,” accessed May 26, 2021, http://www.gov.sz/index.php/latest-news/204-latest-news/2408-national-emergency-management-coordination-structures-2. [↑](#footnote-ref-44)
45. “NATIONAL EMERGENCY MANAGEMENT COORDINATION STRUCTURES.” [↑](#footnote-ref-45)
46. “Stakeholder Engagement Plan (SEP),” n.d. [↑](#footnote-ref-46)
47. “ESwatini Reports First Confirmed Case of Coronavirus,” accessed May 26, 2021, https://ewn.co.za/2020/03/14/eswatini-reports-first-confirmed-case-of-coronavirus. [↑](#footnote-ref-47)
48. Mekonnen Hailemariam Zikargae, “Covid-19 in Ethiopia: Assessment of How the Ethiopian Government Has Executed Administrative Actions and Managed Risk Communications and Community Engagement,” *Risk Management and Healthcare Policy* 13 (2020): 2803–10, https://doi.org/10.2147/RMHP.S278234. [↑](#footnote-ref-48)
49. Zikargae. [↑](#footnote-ref-49)
50. “Ethiopia: COVID-19 Humanitarian Impact - Situation Update No. 01, As of 31 March 2020 - Ethiopia | ReliefWeb,” accessed May 26, 2021, https://reliefweb.int/report/ethiopia/ethiopia-covid-19-humanitarian-impact-situation-update-no-01-31-march-2020. [↑](#footnote-ref-50)
51. Zikargae, “Covid-19 in Ethiopia: Assessment of How the Ethiopian Government Has Executed Administrative Actions and Managed Risk Communications and Community Engagement.” [↑](#footnote-ref-51)
52. “UNDP Gabon,” n.d., 10027. [↑](#footnote-ref-52)
53. “Gabon: Authorities Confirm First Case of COVID-19 March 13 /Update 2,” accessed May 26, 2021, https://www.garda.com/crisis24/news-alerts/322451/gabon-authorities-confirm-first-case-of-covid-19-march-13-update-2. [↑](#footnote-ref-53)
54. “The Gambia’s Public Health Emergency Operations Center: Building Responsive, Sustainable Institutions for Covid-19 and beyond - Dalberg,” accessed May 26, 2021, https://dalberg.com/our-ideas/the-gambias-public-health-emergency-operations-center-building-responsive-sustainable-institutions-for-covid-19-and-beyond/. [↑](#footnote-ref-54)
55. “Health Alert: The Gambia, Government Announces First COVID-19 Case and Implements Measures to Limit Spread,” accessed May 26, 2021, https://www.osac.gov/Content/Report/3a590ebb-0e75-4928-b45e-183acdd88e78. [↑](#footnote-ref-55)
56. “The Gambia’s Public Health Emergency Operations Center: Building Responsive, Sustainable Institutions for Covid-19 and beyond - Dalberg.” [↑](#footnote-ref-56)
57. Joseph Antwi-Boasiako et al., “Policy Responses to Fight Covid-19; the Case of Ghana,” *Revista de Administracao Publica* 55, no. 1 (March 5, 2021): 122–39, https://doi.org/10.1590/0034-761220200507. [↑](#footnote-ref-57)
58. “Guinea: ‘Knowledge Comes from Experience.’ How the Lessons Learned from Combating Ebola Led to a Quick Response from the Very Start of the COVID-19 Epidemic,” accessed May 26, 2021, https://blogs.worldbank.org/nasikiliza/guinea-knowledge-comes-experience-how-lessons-learned-combating-ebola-led-quick-response. [↑](#footnote-ref-58)
59. “UNDP Guinea,” 2020, 10027. [↑](#footnote-ref-59)
60. “Guinea: Authorities Confirm First Case of COVID-19 March 13,” accessed May 26, 2021, https://www.garda.com/crisis24/news-alerts/322511/guinea-authorities-confirm-first-case-of-covid-19-march-13. [↑](#footnote-ref-60)
61. “Lessons from Guinea, Ebola to COVID-19,” accessed May 26, 2021, https://www.sfcg.org/guineapostebola/. [↑](#footnote-ref-61)
62. “Stakeholder Engagement Plan (SEP) Guinea Bissau COVID-19 Emergency Response Project (P174243),” n.d. https://ewsdata.rightsindevelopment.org/files/documents/43/WB-P174243_5qeOkoO.pdf [↑](#footnote-ref-62)
63. “Guinea-Bissau: Country Confirms First Cases of COVID-19 March 25,” accessed May 26, 2021, https://www.garda.com/crisis24/news-alerts/326431/guinea-bissau-country-confirms-first-cases-of-covid-19-march-25. [↑](#footnote-ref-63)
64. “(No Title),” accessed May 26, 2021, https://thinkwell.global/wp-content/uploads/2020/05/COVID-19-Kenya-Update-Dec2020-final.pdf. [↑](#footnote-ref-64)
65. “(No Title),” accessed May 26, 2021, https://documents1.worldbank.org/curated/en/240741588234502363/pdf/Project-Information-Document-Lesotho-COVID-19-Emergency-Preparedness-and-Response-Project-P173939.pdf. [↑](#footnote-ref-65)
66. “Three Ways Lesotho’s Past Experience with Disasters Strengthen COVID-19 Response,” accessed May 26, 2021, https://blogs.worldbank.org/nasikiliza/three-ways-lesothos-past-experience-disasters-strengthen-covid-19-response. [↑](#footnote-ref-66)
67. “Lesotho: Authorities Confirm First COVID-19 Case May 13 /Update 3 | Crisis24,” accessed May 26, 2021, https://crisis24.garda.com/insights-intelligence/intelligence/risk-alerts/a8l6rnagpot2owmgj/lesotho-authorities-confirm-first-covid-19-case-may-13-update-3. [↑](#footnote-ref-67)
68. “Three Ways Lesotho’s Past Experience with Disasters Strengthen COVID-19 Response.” [↑](#footnote-ref-68)
69. “COVID-19 - Response from Liberia | African Regional Organisation of the International Trade Union Confederation,” accessed May 26, 2021, https://www.ituc-africa.org/COVID-19-Response-from-Liberia.html. [↑](#footnote-ref-69)
70. “Liberia: First COVID-19 Case Confirmed March 16,” accessed May 26, 2021, https://www.garda.com/crisis24/news-alerts/323506/liberia-first-covid-19-case-confirmed-march-16. [↑](#footnote-ref-70)
71. “Liberia: Ebola Contact Tracing Lessons Inform COVID-19 Response | | UN News,” accessed May 26, 2021, https://news.un.org/en/story/2020/04/1062582. [↑](#footnote-ref-71)
72. “COVID-19 Situation Report, Madagascar | Funding Overview UNICEF Madagascar Has Estimated Its Initial Needs at USD 7 Million for Four Month,” 2020, https://www.covid19mg.org/. [↑](#footnote-ref-72)
73. Michelle V. Evans et al., “Reconciling Model Predictions with Low Reported Cases of COVID-19 in Sub-Saharan Africa: Insights from Madagascar,” *Global Health Action* 13, no. 1 (December 31, 2020), https://doi.org/10.1080/16549716.2020.1816044. [↑](#footnote-ref-73)
74. “NATIONAL COVID-19 PREPAREDNESS AND RESPONSE PLAN The Republic of Malawi Ministry of Disaster Management Affairs and Public Events Ministry of Health (Developed in Collaboration with UN Humanitarian Country Team and Partners) Photo Credit: MoH,” n.d. http://extwprlegs1.fao.org/docs/pdf/mlw196187.pdf [↑](#footnote-ref-74)
75. “NATIONAL COVID-19 PREPAREDNESS AND RESPONSE PLAN The Republic of Malawi Ministry of Disaster Management Affairs and Public Events Ministry of Health (Developed in Collaboration with UN Humanitarian Country Team and Partners) Photo Credit: MoH.” http://extwprlegs1.fao.org/docs/pdf/mlw196187.pdf [↑](#footnote-ref-75)
76. “Malawi: First COVID-19 Cases Confirmed April 2 /Update 2,” accessed May 27, 2021, https://www.garda.com/crisis24/news-alerts/328736/malawi-first-covid-19-cases-confirmed-april-2-update-2. [↑](#footnote-ref-76)
77. “Mali’s Response to the COVID-19 Pandemic | African Regional Organisation of the International Trade Union Confederation,” accessed May 27, 2021, http://www.ituc-africa.org/Mali-s-response-to-the-COVID-19-pandemic.html. [↑](#footnote-ref-77)
78. “UNICEF Emergency Specialist (NOC) - Coordination of UNICEF Mali Emergency Response to the Covid- Epidemic, TA ( Days) Bamako, Mali,” accessed May 27, 2021, https://www.unjobnet.org/jobs/detail/22789728. [↑](#footnote-ref-78)
79. “Mali’s Response to the COVID-19 Pandemic | African Regional Organisation of the International Trade Union Confederation.” [↑](#footnote-ref-79)
80. “Mali’s Response to the COVID-19 Pandemic | African Regional Organisation of the International Trade Union Confederation.” [↑](#footnote-ref-80)
81. Seydou Doumbia et al., “Coordinating the Research Response to COVID-19: Mali’s Approach,” *Health Research Policy and Systems* (BioMed Central Ltd, September 17, 2020), https://doi.org/10.1186/s12961-020-00623-8. [↑](#footnote-ref-81)
82. “IFRC Review of Emergency Decrees: Mauritania v1.0 6 04 20,” accessed May 27, 2021, http://fr.alakhbar.info/15444-0-Nouakchott-Le-croissant-rouge-sensibilise-sur-le-Covid-19.html. [↑](#footnote-ref-82)
83. “Mauritania: Government Confirm First COVID-19 Case March 13 /Update 1,” accessed May 27, 2021, https://www.garda.com/crisis24/news-alerts/322831/mauritania-government-confirm-first-covid-19-case-march-13-update-1. [↑](#footnote-ref-83)
84. “IFRC Review of Emergency Decrees: Mauritania v1.0 6 04 20.” [↑](#footnote-ref-84)
85. Laurent Musango et al., “Key Success Factors of Mauritius in the Fight against COVID-19 Commentary Handling Editor Seye Abimbola,” *BMJ Global Health* 6 (2021): 5372, https://doi.org/10.1136/bmjgh-2021-005372. [↑](#footnote-ref-85)
86. Musango et al. [↑](#footnote-ref-86)
87. Musango et al. [↑](#footnote-ref-87)
88. “Reaching the Most Vulnerable in the Social Protection Response to the COVID-19 Crises in Mozambique: Opportunities and Challenges,” n.d. https://www.ilo.org/wcmsp5/groups/public/---africa/---ro-abidjan/---ilo-lusaka/documents/publication/wcms_769723.pdf [↑](#footnote-ref-88)
89. “A Comprehensive COVID-19 Response from Government, WHO and Partners Keeps Community Transmission at Bay and Protects Health Services for the Vulnerable | WHO | Regional Office for Africa,” accessed May 27, 2021, https://www.afro.who.int/news/comprehensive-covid-19-response-government-who-and-partners-keeps-community-transmission-bay. [↑](#footnote-ref-89)
90. “A Comprehensive COVID-19 Response from Government, WHO and Partners Keeps Community Transmission at Bay and Protects Health Services for the Vulnerable | WHO | Regional Office for Africa.” [↑](#footnote-ref-90)
91. “Niger Coronavirus (COVID-19) Situation Report #01 (Reporting Period: 6 to 12 April 2020) - Niger | ReliefWeb,” accessed May 27, 2021, https://reliefweb.int/report/niger/niger-coronavirus-covid-19-situation-report-01-reporting-period-6-12-april-2020. [↑](#footnote-ref-91)
92. “Niger Coronavirus (COVID-19) Situation Report #01 (Reporting Period: 6 to 12 April 2020) - Niger | ReliefWeb.” [↑](#footnote-ref-92)
93. Chioma Dan-Nwafor et al., “Nigeria’s Public Health Response to the COVID-19 Pandemic: January to May 2020,” *Journal of Global Health* 10, no. 2 (December 1, 2020), https://doi.org/10.7189/JOGH.10.020399. [↑](#footnote-ref-93)
94. Dan-Nwafor et al. [↑](#footnote-ref-94)
95. Dan-Nwafor et al. [↑](#footnote-ref-95)
96. C. C. Etteh, M. P. Adoga, and C. C. Ogbaga, “COVID-19 Response in Nigeria: Health System Preparedness and Lessons for Future Epidemics in Africa,” *Ethics, Medicine and Public Health* 15 (October 1, 2020), https://doi.org/10.1016/j.jemep.2020.100580. [↑](#footnote-ref-96)
97. Naz Karim et al., “Lessons Learned from Rwanda: Innovative Strategies for Prevention and Containment of COVID-19,” *Annals of Global Health* 87, no. 1 (February 25, 2021): 1–9, https://doi.org/10.5334/aogh.3172. [↑](#footnote-ref-97)
98. “COVID-19 in Rwanda: A Country’s Response | WHO | Regional Office for Africa,” accessed May 27, 2021, https://www.afro.who.int/news/covid-19-rwanda-countrys-response. [↑](#footnote-ref-98)
99. Karim et al., “Lessons Learned from Rwanda: Innovative Strategies for Prevention and Containment of COVID-19.” [↑](#footnote-ref-99)
100. “UN Volunteers Fighting COVID-19 in São Tomé and Príncipe | UNV,” accessed May 27, 2021, https://www.unv.org/Success-stories/UN-Volunteers-fighting-COVID-19-São-Tomé-and-Príncipe. [↑](#footnote-ref-100)
101. “São Tomé and Príncipe: Authorities Confirm First COVID-19 Cases April 6 /Update 1,” accessed May 27, 2021, https://www.garda.com/crisis24/news-alerts/329881/sao-tome-and-principe-authorities-confirm-first-covid-19-cases-april-6-update-1. [↑](#footnote-ref-101)
102. “How Senegal Has Set the Standard on COVID-19 | Devex,” accessed May 27, 2021, https://www.devex.com/news/how-senegal-has-set-the-standard-on-covid-19-98266. [↑](#footnote-ref-102)
103. “How Senegal Has Set the Standard on COVID-19 | Devex.” [↑](#footnote-ref-103)
104. Rdge / Ahhd, / Ecgf, and / Rdgn, “AFRICAN DEVELOPMENT BANK SEYCHELLES COVID-19 CRISIS RESPONSE BUDGET SUPPORT PROGRAM,” 2020. [↑](#footnote-ref-104)
105. Ahhd, Ecgf, and Rdgn. [↑](#footnote-ref-105)
106. “Sierra Leone Institutes Additional COVID-19 Preparedness and Response Measures | WHO | Regional Office for Africa,” accessed May 27, 2021, https://www.afro.who.int/news/sierra-leone-institutes-additional-covid-19-preparedness-and-response-measures. [↑](#footnote-ref-106)
107. “Sierra Leone Confirms First Case of COVID-19 | WHO | Regional Office for Africa,” accessed May 27, 2021, https://www.afro.who.int/news/sierra-leone-confirms-first-case-covid-19. [↑](#footnote-ref-107)
108. “Responding to COVID-19 in Fragile States: The Case of Sierra Leone - IGC,” accessed May 27, 2021, https://www.theigc.org/blog/responding-to-covid-19-in-fragile-states-the-case-of-sierra-leone/. [↑](#footnote-ref-108)
109. “COVID-19 National Response Coordination | STIP COVID-19 Watch | STIP Compass,” accessed May 27, 2021, https://stip.oecd.org/covid/policy-initiatives/covid%2Fdata%2FpolicyInitiatives%2F810. [↑](#footnote-ref-109)
110. Devanand Moonasar et al., “COVID-19: Lessons and Experiences from South Africa’s First Surge,” *BMJ Global Health* 6, no. 2 (February 24, 2021): e004393, https://doi.org/10.1136/bmjgh-2020-004393. [↑](#footnote-ref-110)
111. “FIRST CASE OF COVID-19 CORONAVIRUS REPORTED IN SA | NICD,” accessed May 27, 2021, https://www.nicd.ac.za/first-case-of-covid-19-coronavirus-reported-in-sa/. [↑](#footnote-ref-111)
112. Moonasar et al., “COVID-19: Lessons and Experiences from South Africa’s First Surge.” [↑](#footnote-ref-112)
113. “The Ministry of Health with Support from WHO and Partners Scale-up Response to COVID-19 in South Sudan | WHO | Regional Office for Africa,” accessed May 27, 2021, https://www.afro.who.int/news/ministry-health-support-who-and-partners-scale-response-covid-19-south-sudan. [↑](#footnote-ref-113)
114. “South Sudan Confirms First Case of COVID-19 | WHO | Regional Office for Africa,” accessed May 27, 2021, https://www.afro.who.int/news/south-sudan-confirms-first-case-covid-19. [↑](#footnote-ref-114)
115. UNDP, “UNDP Togo,” 2020, 10027. [↑](#footnote-ref-115)
116. UNDP. [↑](#footnote-ref-116)
117. “U G A N D A R E P O R T 2 Uganda’s Emergency Response to the COVID-19 Pandemic: A Case Study,” 2020, www.thinkwell.global. https://thinkwell.global/wp-content/uploads/2020/09/Uganda-COVID-19-Case-Study-_18-Sept-20201.pdf [↑](#footnote-ref-117)
118. “Coordination Driven Governance Structure in Responding to COVID-19 in Tanzania - Strategic Purchasing Africa Resource Centre (SPARC),” accessed May 27, 2021, https://sparc.africa/2020/10/coordination-driven-governance-structure-in-responding-to-covid-19-in-tanzania/. [↑](#footnote-ref-118)
119. Clifford Silver Tarimo and Jian Wu, “The First Confirmed Case of COVID-19 in Tanzania: Recommendations Based on Lesson Learned from China,” *Tropical Medicine and Health* 48, no. 1 (April 26, 2020): 1–3, https://doi.org/10.1186/s41182-020-00214-x. [↑](#footnote-ref-119)
120. Tarimo and Wu. [↑](#footnote-ref-120)
121. Edgar Simulundu et al., “First COVID-19 Case in Zambia — Comparative Phylogenomic Analyses of SARS-CoV-2 Detected in African Countries,” *International Journal of Infectious Diseases* 102 (January 1, 2021): 455–59, https://doi.org/10.1016/j.ijid.2020.09.1480. [↑](#footnote-ref-121)
122. “(No Title),” accessed May 27, 2021, https://echo.unm.edu/doc/covid/edited_Zambia_COVID-19_Response_AFRO.SF.pdf. [↑](#footnote-ref-122)
123. Simulundu et al., “First COVID-19 Case in Zambia — Comparative Phylogenomic Analyses of SARS-CoV-2 Detected in African Countries.” [↑](#footnote-ref-123)
124. Noah Maulani, Israel Nyaburi Nyadera, and Brian Wandekha, “The Generals and the War against COVID-19: The Case of Zimbabwe,” *Journal of Global Health* 10, no. 2 (December 1, 2020): 1–7, https://doi.org/10.7189/jogh.10.020388. [↑](#footnote-ref-124)
125. “Zimbabwe: First Confirmed Case of COVID-19 March 20 /Update 1,” accessed May 27, 2021, https://www.garda.com/crisis24/news-alerts/325231/zimbabwe-first-confirmed-case-of-covid-19-march-20-update-1. [↑](#footnote-ref-125)
126. Maulani, Nyadera, and Wandekha, “The Generals and the War against COVID-19: The Case of Zimbabwe.” [↑](#footnote-ref-126)
