# Supplementary material for: Coordination mechanisms for COVID-19 in the WHO Regional office for Africa
Source: BMC Health Serv Res. 2022 May 28;22:711. doi: 10.1186/s12913-022-08035-w (PMC9142827; doi:10.1186/s12913-022-08035-w)
Supplement: Supplementary file 2 — Additional file 2. Demographic characteristics of the countries. [file 12913_2022_8035_MOESM2_ESM.docx]

**Additional file 2: Demographic characteristics of the countries**

| **Country** | **Population 2020** | **Sub-region** | **Income categorisation** | **Average life expectancy** | **GDP per capita** | **Human development index** | **Percentage population in the Urban region** | **GHSI** | **Current health expenditure (% of GDP) - 2018** | **Infectious disease resilience index** |
| --- | --- | --- | --- | --- | --- | --- | --- | --- | --- | --- |
| Algeria | 43,851,044 | Northern Africa | Upper middle income | 76.9 | 11,350 | 0.748 | 73.2 | 23.6 | 6.2184267 | 0.496612 |
| Angola | 32,866,272 | Middle Africa | Lower middle income | 61.1 | 6,654 | 0.581 | 66.2 | 25.2 | 2.54900527 | 0.148414 |
| Benin | 12,123,200 | Western Africa | Low income | 61.8 | 3,287 | 0.545 | 47.9 | 28.8 | 2.49141979 | 0.206682 |
| Botswana | 2,351,627 | Southern Africa | Upper middle income | 69.6 | 17,766 | 0.735 | 70.2 | 31.1 | 5.84825039 | 0.548363 |
| Burkina Faso | 20,903,273 | Western Africa | Low income | 61.6 | 2,190 | 0.452 | 30 | 30.1 | 5.62852383 | 0.231504 |
| Burundi | 11,890,784 | Eastern Africa | Low income | 61.6 | 752 | 0.433 | 13.4 | 22.8 | 7.74165535 | 0.354104 |
| Cabo Verde | 555,987 | Western Africa | Lower middle income | 73 | 7,172 | 0.665 | 66.2 | 29.3 | 5.35998297 | 0.486189 |
| Cameroon | 26,545,863 | Middle Africa | Lower middle income | 59.3 | 3,653 | 0.563 | 57 | 34.4 | 3.52989626 | 0.38877 |
| Central African Republic | 4,829,767 | Middle Africa | Low income | 53.3 | 945 | 0.397 | 41.8 | 27.3 | 10.9925308 | 0.000061 |
| Chad | 16,425,864 | Middle Africa | Low income | 54.2 | 1,580 | 0.398 | 23.3 | 28.8 | 4.09880257 | 0.09845 |
| Comoros | 869,601 | Eastern Africa | Low income | 64.3 | 3,081 | 0.554 | 29.2 | 27.2 | 4.58884811 | 0.238068 |
| Congo | 5,518,087 | Middle Africa | Lower middle income | 64.6 | 3,298 | 0.574 | 67.4 | 23.6 | 2.13848233 | 0.268887 |
| Côte d'Ivoire | 26,378,274 | Western Africa | Lower middle income | 57.8 | 5,238 | 0.538 | 51.2 | 35.5 | 4.19003773 | 0.270743 |
| Djibouti | 988,000 | Eastern Africa | Lower middle income | 67.1 | 5,519 | 0.524 | 77.9 | 23.2 | 2.32438445 | 0.297892 |
| DR Congo | 89,561,403 | Middle Africa | Low income | 60.7 | 1,098 | 0.48 | 45 | 26.5 | 3.30075741 | 0.181762 |
| Egypt | 102,334,404 | Northern Africa | Lower middle income | N/A | N/A | N/A | N/A | 39.9 | 4.947577 | 0.530405 |
| Equatorial Guinea | 1,402,985 | Middle Africa | Upper middle income | 58.7 | 18,558 | 0.592 | 72.6 | 16.2 | 2.99830961 | 0.430054 |
| Eritrea | 3,546,421 | Eastern Africa | Low income | 66.3 | N/A | 0.459 | 40.7 | 22.4 | 4.09255791 | 0.252978 |
| Eswatini | 1,160,164 | Southern Africa | Lower middle income | 60.2 | 8,688 | 0.611 | 24 | 31.1 | 6.54034519 | 0.35847 |
| Ethiopia | 114,963,588 | Eastern Africa | Low income | 66.6 | 2,220 | 0.485 | 21.2 | 40.6 | 3.29555655 | 0.382021 |
| Gabon | 2,225,734 | Middle Africa | Upper middle income | 66.5 | 14,870 | 0.703 | 89.7 | 20 | 2.74503946 | 0.40295 |
| Gambia | 2,416,668 | Western Africa | Low income | 62 | 2,207 | 0.496 | 61.9 | 34.2 | 3.09379649 | 0.207809 |
| Ghana | 31,072,940 | Western Africa | Lower middle income | 64.1 | 5,413 | 0.611 | 56.7 | 35.5 | 3.53888083 | 0.462565 |
| Guinea | 13,132,795 | Western Africa | Low income | 61.6 | 2,564 | 0.477 | 36.5 | 32.7 | 3.93172431 | 0.213225 |
| Guinea-Bissau | 1,968,001 | Western Africa | Low income | 58.3 | 1,989 | 0.48 | 43.8 | 20 | 6.99553871 | 0.187841 |
| Kenya | 53,771,296 | Eastern Africa | Lower middle income | 66.7 | 4,330 | 0.601 | 27.5 | 47.1 | 5.16729498 | 0.385436 |
| Lesotho | 2,142,249 | Southern Africa | Lower middle income | 54.3 | 2,768 | 0.527 | 28.6 | 30.2 | 9.28354836 | 0.34486 |
| Liberia | 5,057,681 | Western Africa | Low income | 64.1 | 1,428 | 0.48 | 51.6 | 35.1 | 6.73955679 | 0.213114 |
| Libya | 6,871,292 | Northern Africa | Upper middle income | 72.9 | 15,174 | 0.724 | 80.4 | 25.7 |  | 0.493272 |
| Madagascar | 27,691,018 | Eastern Africa | Low income | 67 | 1,646 | 0.528 | 37.9 | 40.1 | 4.78956461 | 0.170787 |
| Malawi | 19,129,952 | Eastern Africa | Low income | 64.3 | 1,060 | 0.483 | 17.2 | 28 | 9.33423615 | 0.279987 |
| Mali | 20,250,833 | Western Africa | Low income | 59.3 | 2,327 | 0.434 | 43.1 | 29 | 3.8849268 | 0.184254 |
| Mauritania | 4,649,658 | Western Africa | Lower middle income | 64.9 | 5,197 | 0.546 | 54.5 | 27.5 | 4.58039093 | 0.107294 |
| Mauritius | 1,271,768 | Eastern Africa | Upper middle income | 75 | 22,989 | 0.804 | 40.8 | 34.9 | 5.82624388 | 0.107294 |
| Morocco | 36,910,560 | Northern Africa | Lower middle income | 76.7 | 7,515 | 0.686 | 63 | 43.7 | 5.31261539 | 0.569769 |
| Mozambique | 31,255,435 | Eastern Africa | Low income | 60.9 | 1,280 | 0.456 | 36.5 | 28.1 | 8.17430115 | 0.262501 |
| Namibia | 2,540,905 | Southern Africa | Upper middle income | 63.7 | 9,637 | 0.646 | 51 | 35.6 | 7.95077181 | 0.490478 |
| Niger | 24,206,644 | Western Africa | Low income | 62.4 | 1,219 | 0.394 | 16.5 | 32.2 | 7.33380175 | 0.166531 |
| Nigeria | 206,139,589 | Western Africa | Lower middle income | 54.7 | 5,135 | 0.539 | 51.2 | 37.8 | 3.88990784 | 0.270681 |
| Rwanda | 12,952,218 | Eastern Africa | Low income | 69 | 2,226 | 0.543 | 17.3 | 34.2 | 7.54268694 | 0.3553 |
| Sao Tome & Principe | 219,159 | Middle Africa | Lower middle income | 70.4 | 3,964 | 0.625 | 73.6 | 17.7 | 6.26645994 | 0.223256 |
| Senegal | 16,743,927 | Western Africa | Low income | 67.9 | 3,395 | 0.512 | 47.7 | 37.9 | 3.97799659 | 0.329156 |
| Seychelles | 98,347 | Eastern Africa | High income | 73.4 | 29,056 | 0.796 | 57.1 | 31.9 | 5.11036491 | 0.628108 |
| Sierra Leone | 7,976,983 | Western Africa | Low income | 54.7 | 1,718 | 0.452 | 42.5 | 38.2 | 16.0630798 | 0.223397 |
| Somalia | 15,893,222 | Eastern Africa | Low income |  |  |  |  | 16.6 |  | 0 |
| South Africa | 59,308,690 | Southern Africa | Upper middle income | 64.1 | 12,482 | 0.709 | 66.9 | 54.8 | 8.25342083 | 0.697292 |
| South Sudan | 11,193,725 | Eastern Africa | Low income | 57.9 | N/A | 0.433 | 19.9 | 21.7 | 6.40037966 | 0.100836 |
| Sudan | 43,849,260 | Northern Africa | Lower middle income | 65.3 | 3,958 | 0.51 | 34.9 | 26.2 | 4.51304483 | 0.29158 |
| Tanzania | 59,734,218 | Eastern Africa | Lower middle income | 65.5 | 2,660 | 0.529 | 34.5 | 36.4 | 3.62868047 | 0.340445 |
| Togo | 8,278,724 | Western Africa | Low income | 61 | 1,596 | 0.515 | 42.2 | 32.5 | 6.16532087 | 0.259396 |
| Tunisia | 11,818,619 | Northern Africa | Lower middle income | 76.7 | 10,756 | 0.74 | 69.3 | 33.7 | 7.29209566 | 0.535451 |
| Uganda | 45,741,007 | Eastern Africa | Low income | 63.4 | 2,181 | 0.544 | 24.4 | 44.3 | 6.52623558 | 0.36585 |
| Zambia | 18,383,955 | Eastern Africa | Lower middle income | 63.9 | 3,479 | 0.584 | 44.1 | 28.7 | 4.93484306 | 0.420459 |
| Zimbabwe | 14,862,924 | Eastern Africa | Low income | 61.5 | 2,836 | 0.571 | 32.2 | 38.2 | 4.73433113 | 0.337478 |
| SSA | 1,078 million |  |  | 61.2 | 3,988 | 0.541 | 40.2 | N/A | 5.09403365 |  |
| Global | 7,792 million |  |  | 72.6 | 17,948 | 0.731 | 55.3 | N/A | 9.85695151 |  |
