# Supplementary material for: Coordination mechanisms for COVID-19 in the WHO Regional office for Africa
Source: BMC Health Serv Res. 2022 May 28;22:711. doi: 10.1186/s12913-022-08035-w (PMC9142827; doi:10.1186/s12913-022-08035-w)
Supplement: Supplementary file 3 — Additional file 3. Correlation matrix for all independent variables. [file 12913_2022_8035_MOESM3_ESM.docx]

**Additional file 3: Correlation matrix for all independent variables**

**a) correlation matrix for all independent variables**

|  | **Income category** | **Life expectancy** | **gdp_capita** | **hdi** | **urb_pop** | **ghsi** | **health_exp** | **idi** |
| --- | --- | --- | --- | --- | --- | --- | --- | --- |
| **Income category** | 1 |  |  |  |  |  |  |  |
| **Life expectancy** | -0.4419 | 1 |  |  |  |  |  |  |
| **gdp_capita** | -0.8794 | 0.4947 | 1 |  |  |  |  |  |
| **hdi** | -0.8684 | 0.6956 | 0.8392 | 1 |  |  |  |  |
| **urb_pop** | -0.6157 | 0.2706 | 0.4953 | 0.572 | 1 |  |  |  |
| **ghsi** | 0.0499 | 0.032 | -0.062 | 0.0611 | -0.2779 | 1 |  |  |
| **health_exp** | 0.138 | -0.2011 | -0.133 | -0.1816 | -0.3066 | 0.1456 | 1 |  |
| **idi** | -0.627 | 0.4182 | 0.512 | 0.6349 | 0.2965 | 0.2761 | -0.0222 | 1 |

**b) correlation matrix for the chosen variables**

|  | **Life expectancy** | **gdp_capita** | **hdi** | **urb_pop** | **ghsi** | **health_exp** | **idi** |
| --- | --- | --- | --- | --- | --- | --- | --- |
| **Life expectancy** | 1 |  |  |  |  |  |  |
| **gdp_capita** | 0.4947 | 1 |  |  |  |  |  |
| **hdi** | 0.6956 | 0.8392 | 1 |  |  |  |  |
| **urb_pop** | 0.2706 | 0.4953 | 0.572 | 1 |  |  |  |
| **ghsi** | 0.032 | -0.062 | 0.0611 | -0.2779 | 1 |  |  |
| **health_exp** | -0.2011 | -0.133 | -0.1816 | -0.3066 | 0.1456 | 1 |  |
| **idi** | 0.4182 | 0.512 | 0.6349 | 0.2965 | 0.2761 | -0.0222 | 1 |
